# Novel benzimidazole-1, 3, 4-thiadiazole derivatives as casein kinase-2 inhibitors: synthesis, in vitro and in silico investigations

**DOI:** 10.1186/s13065-025-01532-z

**Published:** 2025-06-09

**Authors:** N. Senthilkumar, S. Sarveswari, Prafulla Choudhari, Somdatta Chaudhari, Imadul Islam, Yasinalli Tamboli, V. Vijayakumar

**Affiliations:** 1 Department of Chemistry, Vellore Institute of Technology, Vellore, Tamil Nadu 632 014 India; 2 Department of Pharmaceutical Chemistry, Bharati Vidyapeeth College of Pharmacy, Kolhapur, India; 3 Department of Pharmaceutical Chemistry, PES’s Modern College of Pharmacy, Pune, Maharashtra 411 044 India; 4 King Abdullah International Medical Research Center (KAIMRC), Ministry of National Guard-Health Affairs, King Saud Bin Abdulaziz University for Health Sciences, 14811 Riyadh, Saudi Arabia

**Keywords:** Benzimidazole-thiadiazole derivatives, Synthesis, Docking, Cytotoxicity, HeLa cell line

## Abstract

A new set of benzimidazole-thiadiazole derivatives has been designed and synthesized using 2-(chloromethyl)-1*H*-benzo[d]imidazole (**1**). Compounds **4a-m** were achieved by amide bond formation using acid chlorides and pyridine in 1, 2-dichloroethane. The newly synthesized compounds were characterized and subjected to cytotoxicity studies against HeLa cells. Compounds **4c**, **4 d**, and **4e** exhibited good inhibitory activity with IC_50_ values of 15, 25, and 25 µM, respectively. Molecular docking studies were performed to elucidate the binding interactions of compounds **4c** and **4e** with human Casein kinase-2 (CK2). Compound **4c** exhibited maximum cytotoxicity against HeLa cells with the lowest binding energy values of -8.61 kcal/mol towards CK2. Compound **4c** underwent molecular dynamics (MD) simulations to assess their stability and interactions within the active site. Density functional theory (DFT) calculations also provided insights into the synthesised compounds' electronic structure, reactivity, and charge distribution.

**Graphical Abstract:**

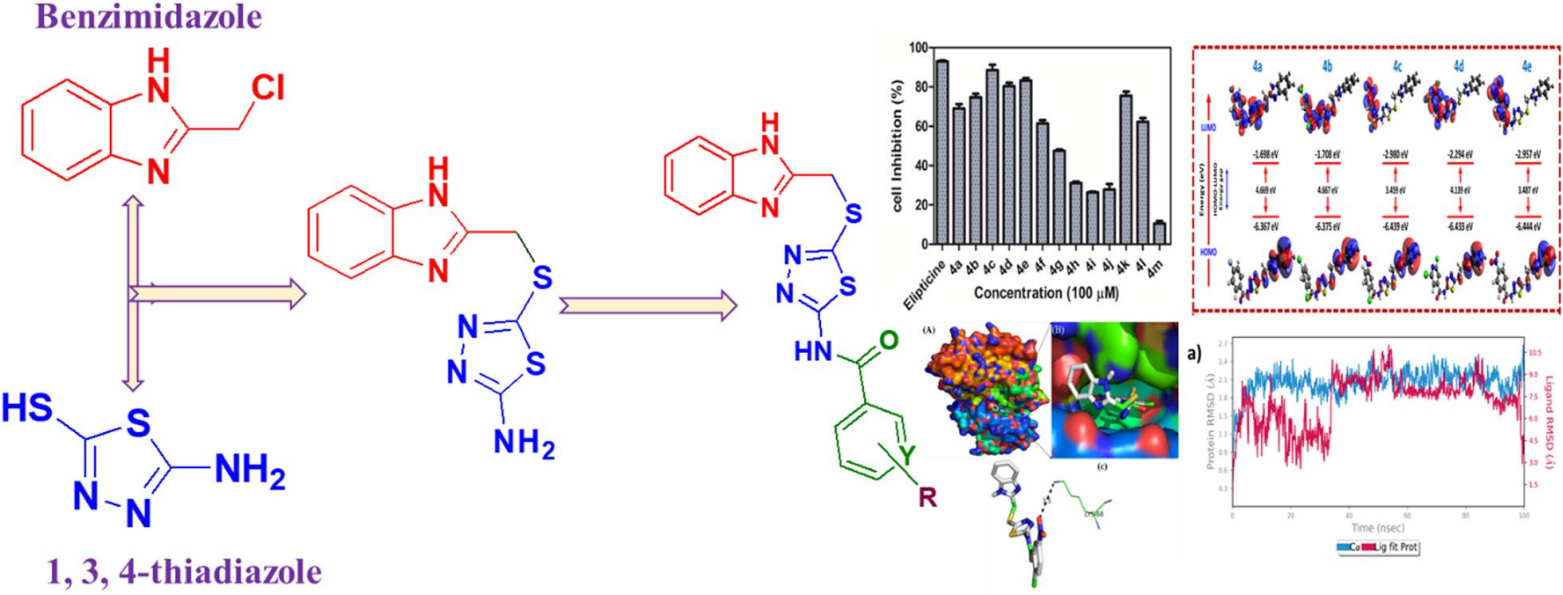

**Supplementary Information:**

The online version contains supplementary material available at 10.1186/s13065-025-01532-z.

## Introduction

The development of novel anticancer drugs remains a critical focus in medicinal chemistry. One major challenge for medicinal chemists is identifying compounds that effectively target the complex biochemical pathways involved in cancer progression. Casein kinase-2 (CK2), a highly conserved serine/threonine kinase, plays a pivotal role in regulating cell growth, proliferation, and survival. Overexpression, dysregulation, and hyperactivation of CK2 have been implicated in various cancers, including head and neck, leukemia, breast, colorectal, renal, lung, and prostate cancers [1]. CK2 has numerous physiological targets, including maintaining cell viability, and has long been recognized for its role in supporting cell growth and proliferation [2]. Furthermore, the downregulation of CK2 activity has been found to induce apoptosis in cancer cells, emphasizing the importance of developing CK2 inhibitors for the treatment of cancers [3]. CK2 is regarded as a druggable protein kinase target, making it a promising candidate for the development of antitumor, antiviral, and anti-inflammatory therapies [4]. Halogenated benzimidazole and benzimidazole compounds have been found to act as CK2 inhibitors [5]. Highly selective and potent CK2 inhibitors have demonstrated broad-spectrum antiproliferative activity, particularly in inflammatory breast cancer cell lines [6].

**Fig. 1:**
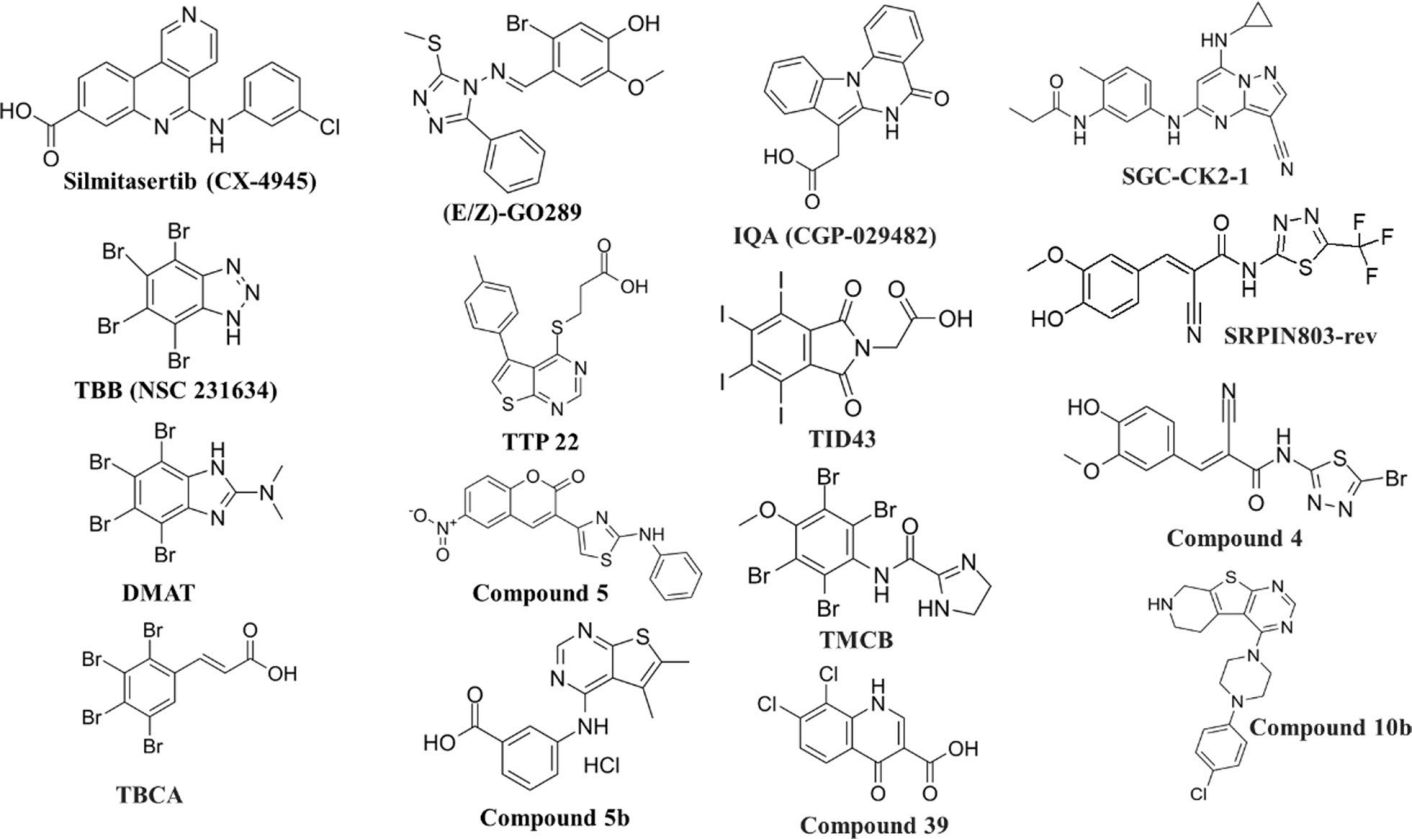
Structures of reported CK2 inhibitors

Casein Kinase 2 (CK2) Inhibitors such as Silmitasertib (CX-4945) [7], (E/Z)-GO289 [8], IQA (CGP-029482) [9], SGC-CK2-1 [10], TBB [11], TTP 22 [12], TID43 [13], DMAT [14], TMCB [15], SRPIN803-rev and Compound 4 [16], Compound 5 [17], Compound 5b [18], TBCA [19], Compound 10b [20], and Compound 39 [21] are a class of small molecules that selectively inhibit the activity of CK2 as shown in Fig. 1.

**Fig. 2:**
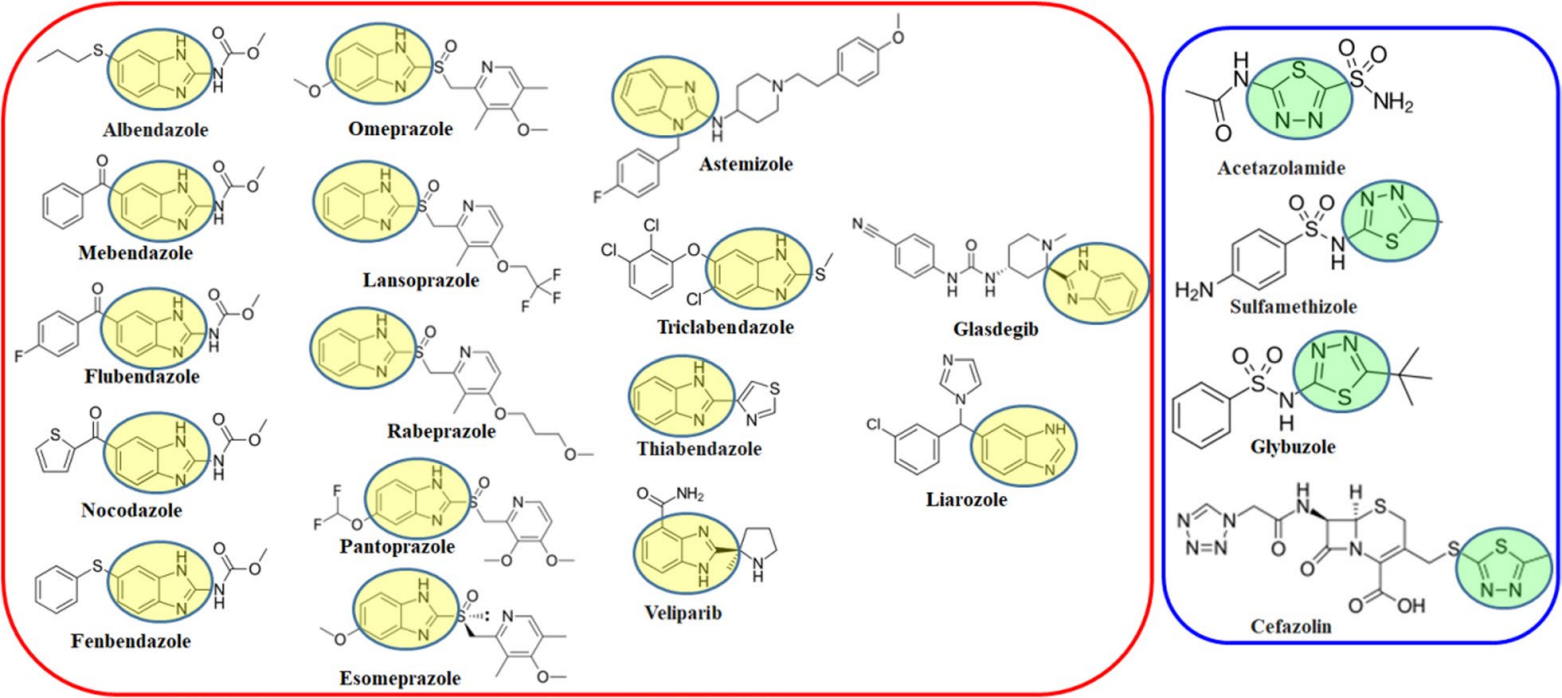
Known molecules with benzimidazole and thiadiazole scaffold

In recent decades, molecular biology and drug development have been involved in the screening of various fused heterocyclic compounds to evaluate anticancer agents. Benzimidazole derivatives are among the most significant fused heterocyclic compounds, serving as structural isosteres of naturally occurring nucleotides. Structurally, it resembles the purine bases of nucleic acids, which enables it to interact effectively with diverse biological targets such as enzymes and receptors. Their capacity to form strong hydrogen bonds and engage in π-π stacking interactions enhances their binding affinity toward biomolecules, making them attractive candidates in drug design [22, 23]. Benzimidazole has played a pivotal role in the discovery of new drugs by offering a unique combination of chemical flexibility (can be easily functionalized at multiple positions allowing chemists to fine-tune solubility, potency, selectivity, and pharmacokinetics) and biological compatibility (high binding affinity and specificity). Several clinically approved drugs are based on benzimidazole frameworks as shown in Fig. 2, highlighting their therapeutic importance. This structural similarity enables them to interact readily with biopolymers within living systems, contributing to their diverse biological activities, including anticancer [24–28], antitumor [29–31], antimicrobial [32, 33], antitubercular [34], and antioxidant [35] activities.

The thiadiazole nucleus, a five-membered heterocyclic ring system containing two nitrogen atoms and one sulfur atom, has emerged as a privileged scaffold in medicinal chemistry. Its inherent high electron density facilitates strong electrostatic and π–π stacking interactions with biological targets, while its planar and chemically stable structure supports optimal receptor binding [36]. Furthermore, the thiadiazole core exhibits dual hydrogen bond donor and acceptor capabilities, enhancing binding affinity and specificity towards diverse biological macromolecules. The structural diversity, chemical flexibility, favorable membrane permeability, metabolic stability, and good oral bioavailability associated with thiadiazole-containing compounds make this scaffold particularly attractive for rational drug design and development [37]. The incorporation of the thiadiazole moiety into pharmacophores has consistently led to improved biological activity, enhanced pharmacokinetic profiles, and broadened therapeutic potential. Numerous thiadiazole derivatives have demonstrated significant pharmacological activities, including antimicrobial [38], anti-cancer [39–41], anti-inflammatory [42], anti-tubercular [43], anti-viral [44], anti-leishmanial [45], and anti-epileptic agents [46]. These versatile attributes underscore the vital role of the thiadiazole scaffold as a cornerstone in modern drug discovery efforts.

Amide bond linkages are fundamental in drug design and discovery, primarily because of their ability to provide novelty, structural stability, versatility, and biological activity in therapeutic agents. These linkages, R-CO–NH-R_1_ formed between the carboxyl acid and the amine group [47]. Hence, developing a new class of hybrids that integrate benzimidazole and 1,3,4-thiadiazole pharmacophore is a promising approach for designing effective anticancer agents. Substituting the methyl group of benzimidazole at the 2-position with basic moieties has demonstrated notable anti-tumor activity [30]. The effect of replacing basic moieties such as methyl thio-thiadiazole amides with electron-donating and withdrawing groups, substituted aromatic carbocyclic and heterocyclic amides have been investigated. Several hybrids have been synthesized, subjected to Lipinski's rule, and then to molecular docking studies to find the lead molecule and these results have been described.

**Fig. 3:**
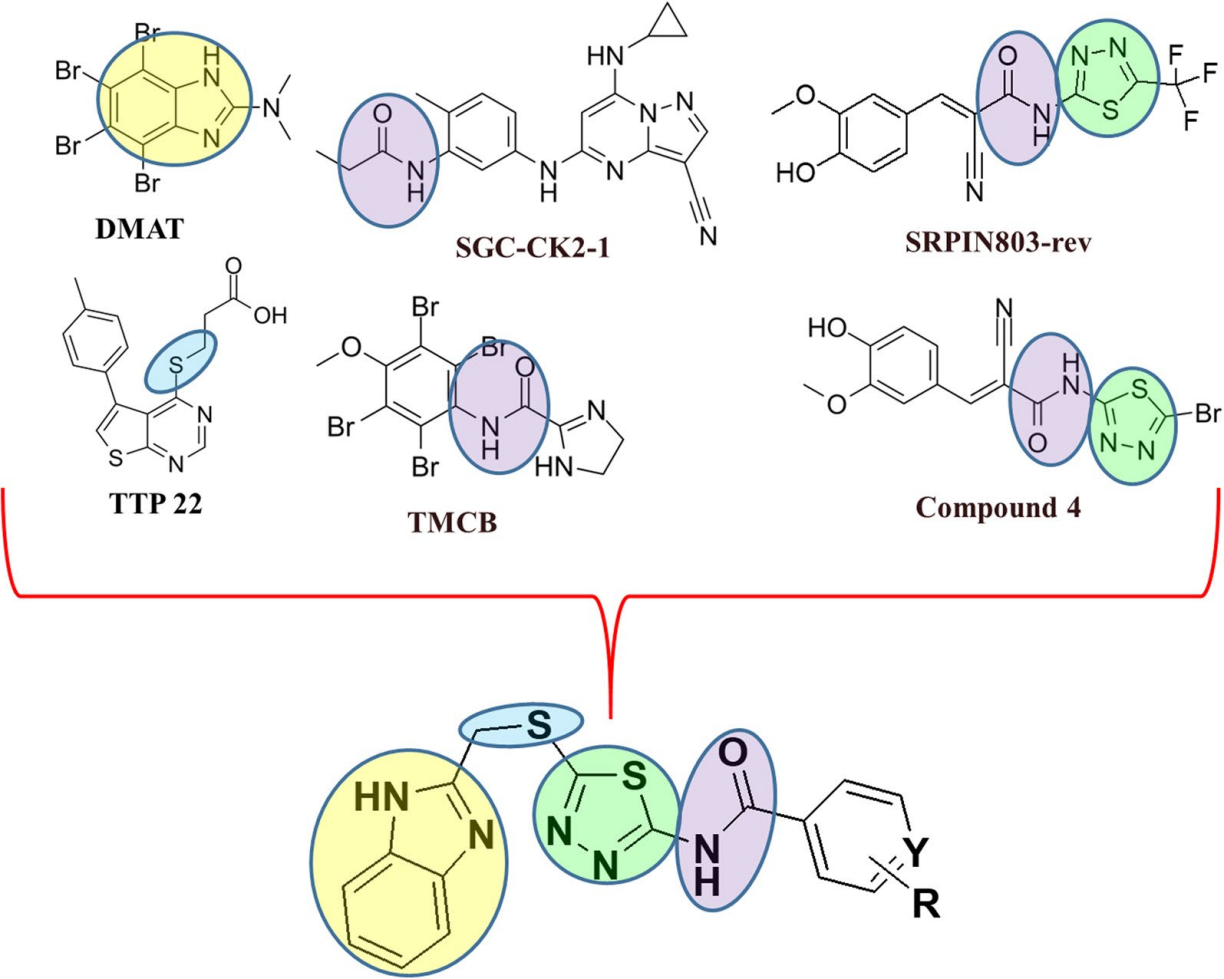
Rational for design of potent CK2 inhibitors

Benzimidazole-thiadiazole hybrids have biological activity that is synergistic when combined and frequently outperforms the effectiveness of each of the individual ingredients. According to structure–activity relationship (SAR) studies, biological activity is greatly impacted by substitution at the 2-position of benzimidazole and the 5-position of thiadiazole, particularly for kinase inhibition. Increased potency and selectivity have been associated with the presence of lipophilic or electron-withdrawing substituents at these locations [48, 49]. The active scaffolds of novel benzimidazole-1,3,4-thiadiazole derivatives aligns closely with the pharmacophoric requirements for potent CK2 inhibitors like benzimidazole in DMAT, thiol linker in TTP22, 1,3,4-thiadiazolet in SRPIN803-rev and Compound 4, and amide bond in TMCB, SGC-CK2-1 as shown in Fig. 3. The dual presence of heteroaromatic structures like benzimidazole and thiadiazole are crucial for biological activity with added advantage of thiol linker and amide bond offers a promising framework for the development of new CDK2-targeting anticancer agents. The goal of adding different substituents to the benzimidazole–1,3,4-thiadiazole scaffold was to introduce novelty and modify important physicochemical and electrical characteristics that are known to affect CK2 inhibition. To investigate binding affinity, both electron-donating and electron-withdrawing groups were included. In order to find substituent patterns that maximize cellular absorption and target selectivity, variation in steric bulk and polarity was also included.

## Results and discussion

**Scheme 1.**
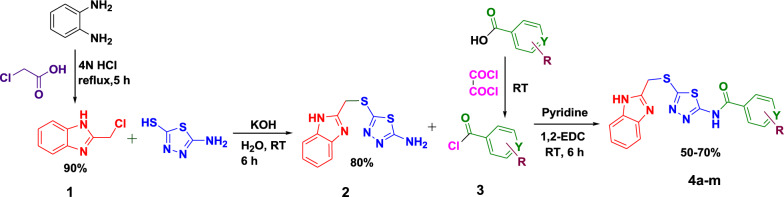
Synthetic route for compounds **4a-m**

The reaction sequences employed for synthesizing N-(5-((1*H*-benzo[d]imidazol-2-yl)methylthio)−1,3,4-thiadiazol-2-yl)-substituted aromatic and heterocyclic amides **4a-m** is outlined in Scheme 1.

**Fig. 4:**
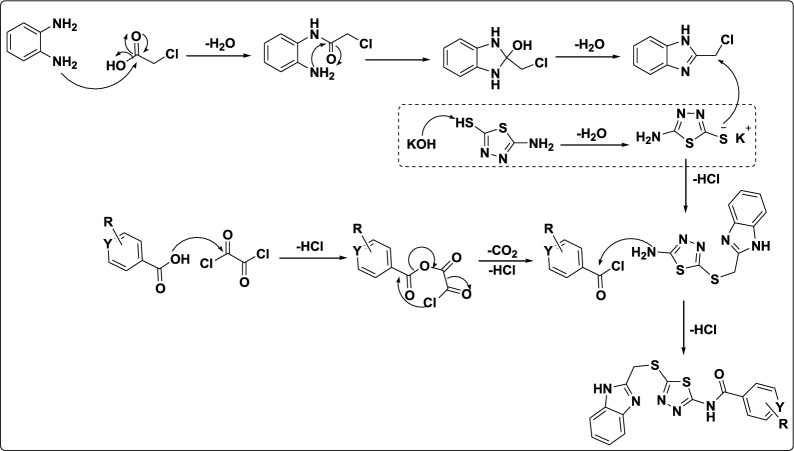
A plausible reaction mechanism for synthesis of compounds **4a-m**

2-(chloromethyl)−1*H*-benzo[d]imidazole (**1**) was obtained in a 90% yield [30] by adding chloroacetic acid to *o*-phenylenediamine in the presence of 4 N HCl. Compound **1** was then treated with 5-amino-1,3,4-thiadiazole-2-thiol and potassium hydroxide in water to afford 5-((1H-benzo[d]imidazol-2-yl)methylthio)−1,3,4-thiadiazol-2-amine (**2**) in 80% yield. A series of coupling reagents and conditions were evaluated to synthesize compounds **4a-m** by combining compound **2** with substituted aryl or heterocyclic acids as shown in Table 1. Most coupling reagents and conditions failed to produce the desired amide bond, highlighting the challenges in activating the carboxylic acid substrate under these conditions. The successful reaction using oxalyl chloride demonstrates the importance of acid chloride intermediates in overcoming steric and electronic barriers to form amide bond. Hence, the derivatives **4a-m** were obtained by treatment of compound **2** with various acid chloride derivatives **3** (prepared in-house) and pyridine as a base in dried 1,2-dichloroethane at room temperature for 6 h. The reagents and reaction conditions were selected to promote high-yielding and efficient product formation with minimum impurities. These conditions were optimized based on literature precedent and screening. 1,2-Dichloroethane was selected as the reaction solvent because it can easily dissolve reagents and reactants, an easy-to-remove boiling point of 84 °C, minimizes interference with reactive electrophiles or catalysts. A plausible reaction mechanism for synthesis of compounds **4a-m** is shown in Fig. 4. The condensation of o-phenylenediamine with chloroacetic acid yields a dihydrobenzimidazole intermediate, which undergoes aromatization via water elimination to form benzimidazole. A thiol nucleophile then displaces the chloro substituent through nucleophilic substitution. Subsequently, a substituted carboxylic acid reacts with oxalyl chloride to form a mixed anhydride, which eliminates CO₂, CO, and HCl to generate the corresponding acid chloride. Nucleophilic attack by an amine on the acid chloride affords an intermediate that transformed to form the amide bond with concurrent elimination of HCl. The structures, IUPAC names and yields (50–70%) of synthesized compounds tabulated in Table S1. Acid chlorides were produced by treating acids with oxalyl chloride at room temperature under a nitrogen atmosphere and used without purification. The structure of all the target compounds **4a-m** were confirmed by the IR, [1]H NMR, [13]C NMR, HRMS and DEPT data.

**Table 1:** Optimization of amide coupling for synthesis compounds **4a-m**

Entry	Reagents/Base/Solvent	Result
1	DIPEA/pyridine and T_3_P in EtOAc [50, 51]	No reaction
2	dicyclohexyl carbodiimide (DCC) in DCM [52]	No reaction
3	Diisopropyl carbodiimide (DIC) in DCM [52]	No reaction
4	1-ethyl-3-(3'-dimethylamino) carbodiimide HCl salt, hydroxybenzotriazole(HOBT), *N,N*-Diisopropylethylamine in DMF [52] or water [47]	No reaction
5	1-[*Bis*(dimethylamino)methylene]−1*H*−1,2,3-triazolo[4,5-b]pyridinium 3-oxid hexafluorophosphate (HATU), *N,N*-Diisopropylethylamine in DMF [52]	No reaction
6	Oxalyl chloride, acid, EDC; amine and pyridine in EDC^a^	50–70%^b^

^a^ Formation of acid chloride followed by amide coupling ^b^ Isolated yield

**Fig. 5:**
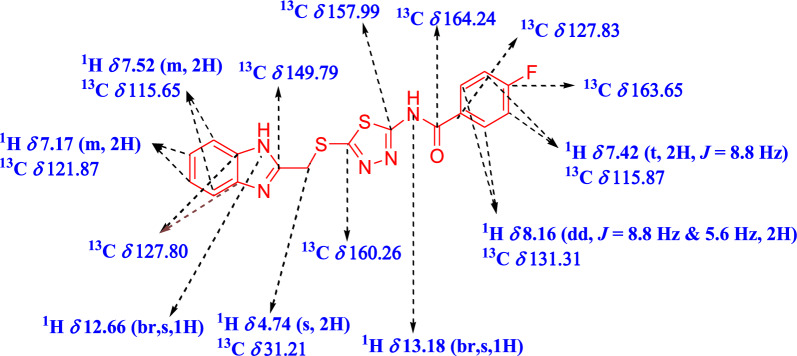
Selected ^1^H, ^13^C NMR chemical shift of compound **4a**

The compounds **4a** was analysed using IR, [1]H NMR, [13]C NMR spectroscopy. The IR spectrum displayed two peaks, one in the range of 1672–1676 cm^−1^ due to the presence of a C = O group, and the other in the range of 1653–1658 cm^−1^ due to the presence of a C = N group. The ^1^H NMR spectrum of compound **4a** was chosen as an example. The –CH protons of benzimidazole resonated at δ 7.17 (m) and 7.52 (m) ppm, while the N–H proton of benzimidazole resonated at δ 12.60 ppm. Additionally, the methylene proton at position-2 of benzimidazole resonated at δ 4.74 ppm. Two signals of phenyl protons were observed at δ 7.42 (t) and 8.16 (dd) ppm. The N–H proton of amide resonated at δ 13.18 (s) ppm, indicating the formation of amide. The ^13^C spectrum of **4a** showed 13 signals as expected. In the DEPT-135 spectrum of compound **4a**, the -CH carbon of benzimidazole was observed at δ 115.65 and 121.87 ppm, while phenyl ring carbons were observed at δ 131.31 ppm in the positive axis. The methylene carbon attached at position-2 of benzimidazole showed a signal at δ 31.21 ppm in the negative axis. The absence of signals in DEPT-135 at δ 127.80, 149.79, 157.99, 160.26 and 163.65 ppm confirmed these carbons as quaternary carbons. These signals are tentatively assigned to benzimidazole ring carbon, carbon at position-2 of benzimidazole, fluorine-substituted carbon in phenyl ring, and thiadiazole ring. The presence of a signal at δ 164.24 ppm confirmed the carbonyl carbon in the ^13^C NMR. The number of protons and carbons corresponding to compound **4a** are shown in Fig. 5.

**Fig. 6:**
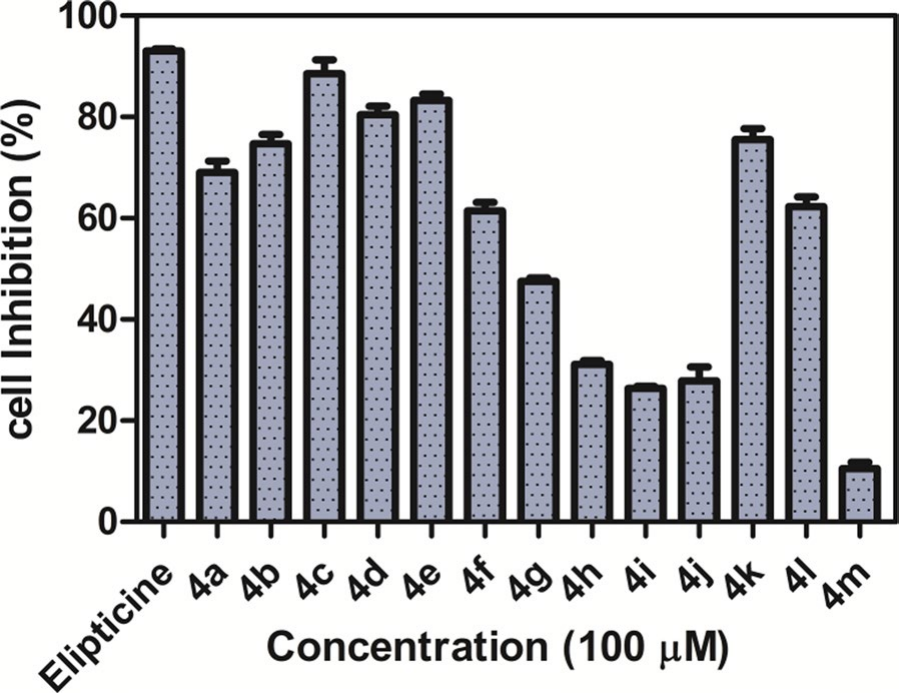
Percentage cell inhibition of compounds **4a-m** on HeLa cell line

Figure 6 and Table 2 showed the percentage cell inhibition at 100 µM concentration and IC_50_ (µM) against cervical cancer cells such as HeLa cells. The HeLa cell line is selected because it provides a human-derived, rapidly proliferating, drug-sensitive, and historically validated model, ideal for initial drug discovery, cytotoxicity assays, and mechanistic studies in cancer research [53]. Among the 13 derivatives, compounds **4c, 4 d** and **4e** exhibited good inhibitory activity (89%, 80% and 83%) respectively like Elipticine 93%. Moreover, Compound **4c (**IC_50_ 15 µM), **4 d (**IC_50_ 25 µM), and **4e (**IC_50_ µM) displayed substantial activity compared with standard drug Elipticine (IC_50_ 9.6 µm). Compound **4a, 4f, 4 k** and **4b** exhibited ~ 60–70% inhibitory activity, whereas remaining compounds **4 h, 4i, 4j** and **4 m** exhibited < 50% inhibitory activity (31%, 28%, 30% and 10% respectively).

**Table 2:** Results of *in-vitro* cytotoxicity against HeLa cell

Entry	Compounds	IC_50_(µM)
1	**4a**	45
2	**4b**	40
3	**4c**	15
4	**4 d**	25
5	**4e**	25
6	**4f**	48
7	**4 g**	58
8	**4 h**	> 100
9	**4i**	> 100
10	**4j**	> 100
11	**4 k**	38
12	**4 l**	42
13	**4 m**	> 100
14	Elipticine	9.81

The compounds numbers are designated in bold

SAR revealed that the *meta*-nitro along with *ortho*-chloro derivative **4c** and *para*-nitro **4e** derivatives exhibited good inhibitory activity. By providing significant electron-withdrawing effects, hydrogen bonding, and advantageous interactions inside the kinase's polar ATP-binding region, nitro groups increase the activity of CK2 inhibitors and improve binding affinity and selectivity. Two nitro group in *meta* position **4 m** was unfavorable for inhibitory activity. *Meta*-trifluoromethyl derivatives **4 k** exhibited good inhibitory activity than *para*-cyano **4 l** derivative. In case of halogen substituted derivatives, *ortho, meta*-dichloro derivative **4b** and 2,6-dichloro isonicotinic derivative **4 d** showed good inhibitory activity. Among the mono substituted chlorine derivatives, *para* position **4f** was more favorable than *ortho* **4 g**. *Para* substituted fluorine derivative **4a** exhibited moderate inhibitory activity. Pyridine derivatives **4 h** and electron donating substituents **4i, 4j** were unfavorable for inhibitory activity. The hydrophobic substituent such as trifluoromethyl, nitro, cyano and halogen groups enhanced inhibition of cell proliferation. However, electron donating substituents are unfavorable.

Both binding affinity and inhibitor selectivity are significantly impacted by the electronic effects of substituents, especially electron-withdrawing groups (EWGs) as opposed to electron-donating groups (EDGs), which have an effect on CK2 inhibition. EWGs are generally associated Improve inhibitor orientation and docking geometry by influencing electronic density at key pharmacophores. EDGs generally activity unless specifically oriented to form stabilizing hydrogen bonding. Electron-rich aromatic scaffolds such as Benzimidazoles and 1,3,4-thiadiazole align well with CK2's flat, narrow ATP pocket by enhancing π–π stacking with hydrophobic residues. The observed IC₅₀ values of **4c** fall within the low micromolar range, comparable to or less than ellipticine, a known cytotoxic agent. While this comparison highlights its promising antiproliferative activity, it is also important to consider clinical relevance. However, further evaluation, including selectivity profiling against normal cells and kinase panels, is necessary to assess therapeutic windows and translational potential.

**Fig. 7:**
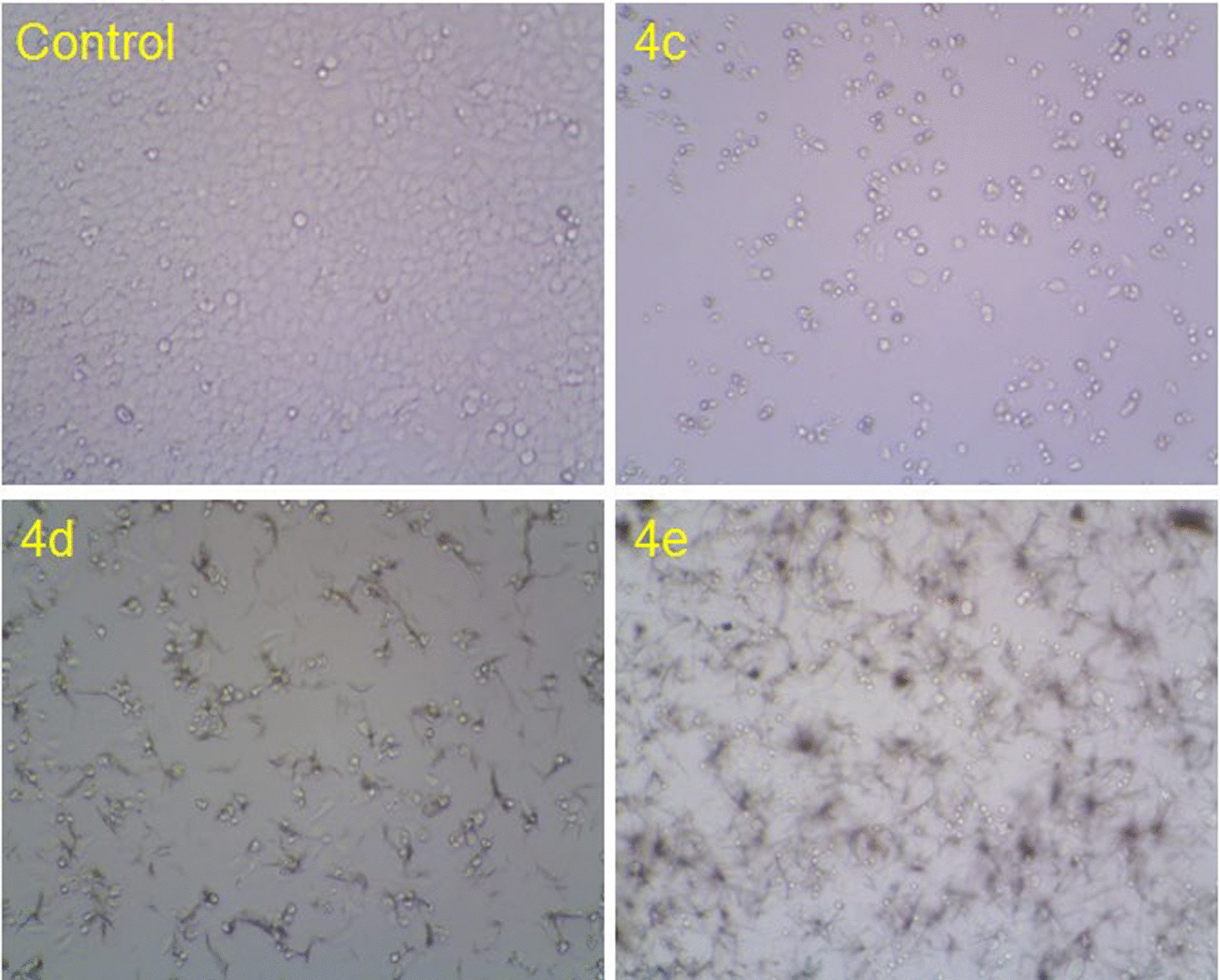
Cytotoxicity study of compounds **4c, 4 d** and **4e** against HeLa cell line

A morphological image of cytotoxicity study of excellent activity **4c, 4 d** and **4e** against HeLa cell line at 100 µM concentration after 48 h showed in Fig. 7. All the compounds were affected the cells by losing its viability as seen by irregular shape morphology compared to control.

### Density functional theory (DFT) study

**Fig. 8:**
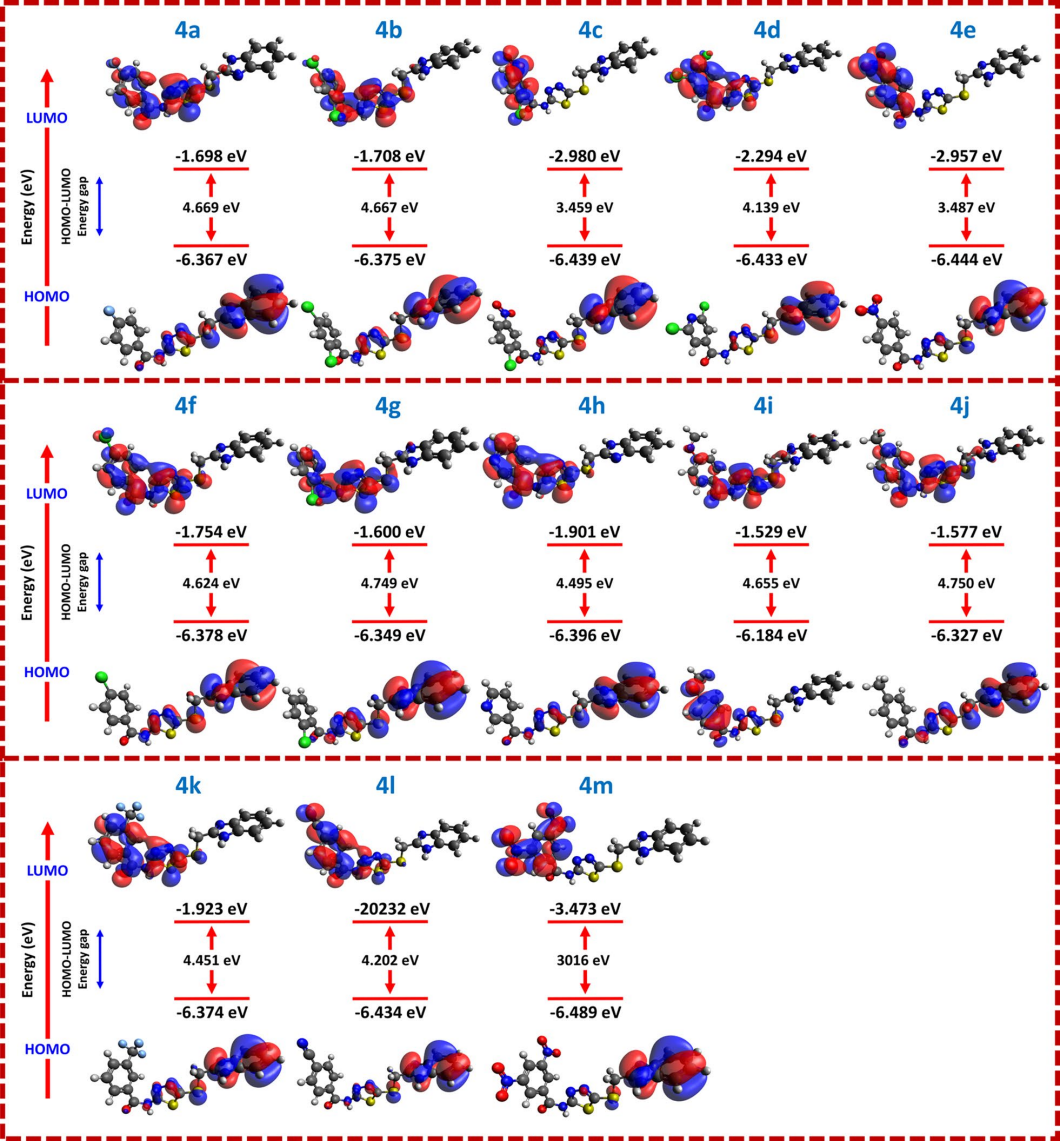
FMO profiles of benzimidazole-1, 3, 4-thiadiazole derivatives (**4a**–**4 m**), showing the HOMO and LUMO distributions, energy values, and HOMO–LUMO energy gaps

The chemical reactivity descriptors of benzimidazole-1,3,4-thiadiazole derivatives (**4a**–**4 m**) were calculated using the DFT method in ORCA. This combination of DFT study, ADMET, and physicochemical screening provided a comprehensive understanding of their potential as drug candidates. The DFT study helped estimate the synthesized compounds'chemical reactivity descriptors and frontier molecular orbitals [54]. The predicted chemical reactivity descriptors and localization of FMOS are presented in Fig. 8 and Table 3. While compound **4 m** demonstrated favorable chemical reactivity regarding electronic structure, compound **4c** was the most active in vitro study with good IC_50_ values. This discrepancy between in silico predictions and experimental results highlights the complexity of drug discovery. Both reactivity and other factors contribute to biological activity. These factors include molecular interactions and stability. The HOMO and LUMO energy levels are key indicators for the ability of a compound to donate and accept electrons. Compound **4 m** exhibited a relatively low HOMO energy (−6.489 eV), suggesting electron-donating solid properties, while **4c** had a comparable HOMO energy of −6.439 eV. The LUMO of **4 m** was −3.473 eV, while the LUMO of **4c** was slightly higher at −2.980 eV. This suggests that **4 m** might be more reactive toward nucleophilic species, whereas **4c**, with a higher LUMO, could interact differently with biological targets. The HOMO–LUMO gap (HLG) of **4c** (3.459 eV) is more significant than that of **4 m** (3.016 eV), indicating that, according to DFT predictions, **4 m** should be more chemically reactive. However, the higher reactivity of **4 m** does not translate to greater in vitro activity. This suggests that other factors may be more significant in determining its biological efficacy [55]. These factors could include the ability of compounds to bind to the target or their stability in a biological environment.

**Table 3:** Energies of chemical reactivity descriptors for synthesized benzimidazole-1, 3, 4-thiadiazole derivatives (**4a**–**4 m**) calculated using the DFT method

Code	HOMO (eV)	LUMO (eV)	HLG (eV)	DM (Debye)	IP (eV)	EA (eV)	χ (eV)	µ (eV)	η (eV)	ω (eV)
**4a**	−6.367	−1.698	4.669	4.265	6.367	1.698	4.033	−4.033	2.335	3.483
**4b**	−6.375	−1.708	4.667	3.469	6.375	1.708	4.042	−4.042	2.334	3.500
**4c**	−6.439	−2.980	3.459	7.661	6.439	2.980	4.710	−4.710	1.730	6.412
**4 d**	−6.433	−2.294	4.139	5.861	6.433	2.294	4.364	−4.364	2.070	4.600
**4e**	−6.444	−2.957	3.487	7.073	6.444	2.957	4.701	−4.701	1.744	6.336
**4f**	−6.378	−1.754	4.624	4.103	6.378	1.754	4.066	−4.066	2.312	3.575
**4 g**	−6.349	−1.600	4.749	2.942	6.349	1.600	3.975	−3.975	2.375	3.326
**4 h**	−6.396	−1.901	4.495	6.196	6.396	1.901	4.149	−4.149	2.248	3.829
**4i**	−6.184	−1.529	4.655	2.866	6.184	1.529	3.857	−3.857	2.328	3.195
**4j**	−6.327	−1.577	4.750	3.983	6.327	1.577	3.952	−3.952	2.375	3.288
**4 k**	−6.374	−1.923	4.451	5.359	6.374	1.923	4.149	−4.149	2.226	3.867
**4 l**	−6.434	−2.232	4.202	6.865	6.434	2.232	4.333	−4.333	2.101	4.468
**4 m**	−6.489	−3.473	3.016	7.436	6.489	3.473	4.981	−4.981	1.508	8.226

The compounds numbers are designated in bold

The reactivity order based on the HOMO–LUMO gap is as follows: 4 m > 4c > 4e > 4 d > 4 h > 4i > 4 k > 4a > 4f > 4 g > 4 l > 4b > 4j. According to this order, **4 m**, with the smallest HOMO–LUMO gap, should theoretically exhibit the highest reactivity, followed by **4c**. However, in vitro studies show that **4c** exhibits superior biological activity to **4 m**. This discrepancy may arise due to the importance of other molecular properties, such as solubility, bioavailability, and target specificity, which influence the actual bioactivity in a cellular environment. Compound **4c** has a slightly higher HOMO–LUMO gap. However, it might interact more effectively with the biological target. This suggests its molecular structure may confer enhanced binding affinity or stability. These effects are not fully captured by the in silico reactivity models. The DFT results provide essential insights into the electronic structure of these compounds. However, these results must be interpreted in conjunction with experimental findings. For instance, the dipole moment (DM) of **4c** (7.661 D) is significantly higher than that of **4 m** (3.016 D). This indicates that **4c** may exhibit stronger intermolecular interactions in polar environments, potentially leading to better solubility and interactions with biological targets. The higher DM of **4c** could also enhance its ability to interact with proteins or nucleic acids, which is critical for its activity in biological systems.

Additionally, **4c** demonstrated the highest DM among the derivatives, which may correlate with its favorable pharmacokinetic properties. Higher DM generally suggests better solubility and permeability in polar environments, potentially explaining the enhanced in vitro activity of **4c**. Compound **4c** exhibited intestinal absorption of 89.062% and favorable distribution characteristics with a low BBB permeability (log BB −1.592). Its log Papp value of 1.114 further supports its potential for good oral bioavailability. Despite its relatively high DM, **4c** showed favorable absorption and permeability, indicating that these physicochemical properties contribute to enhanced in vitro activity. The discrepancy between the reactivity order and experimental results underscores the importance of considering multiple factors, such as molecular interactions, stability, and solubility, when predicting the biological activity of compound **4c**. Future studies should focus on optimizing these compounds by enhancing their target specificity and minimizing potential toxic effects, thus improving their overall drug-likeness and therapeutic potential.

### Molecular docking study

**Fig. 9:**
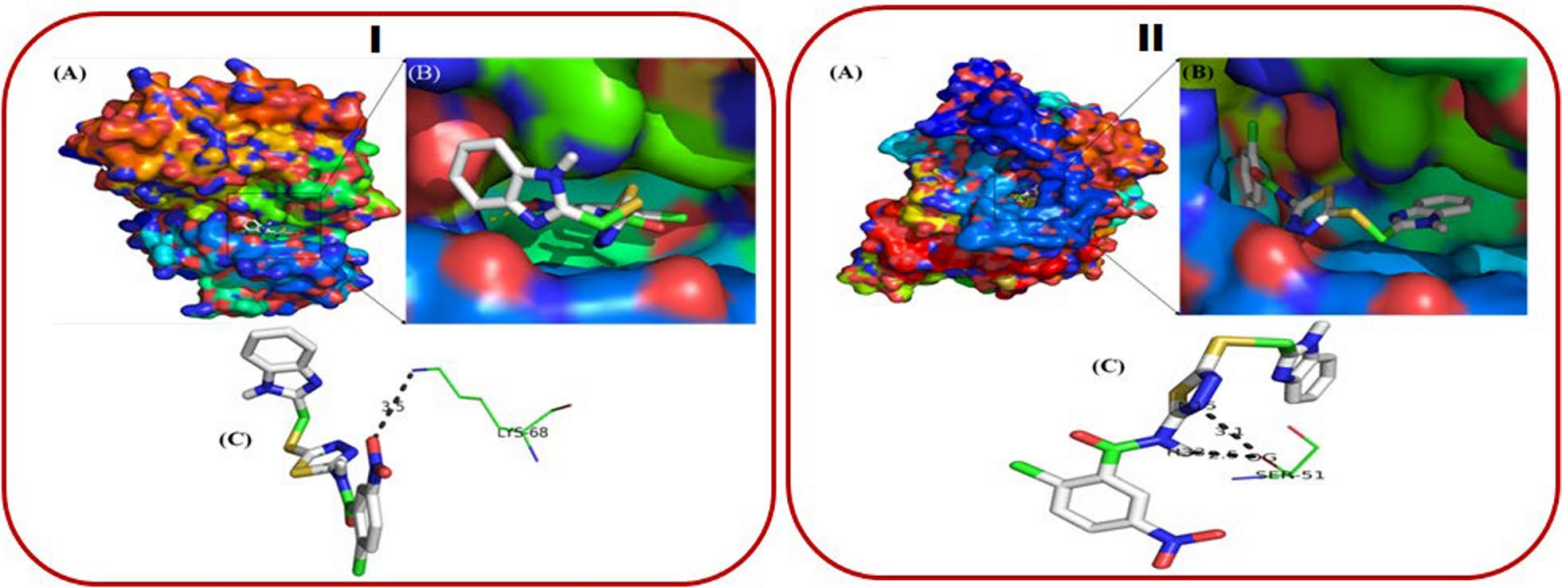
**A** Binding mode of compound **4c** (I) and Compound **4e** (II) into the cavity of CK2. **B** A close-up view of the binding mode. **C** Close-up view of ribbon model

Molecular docking studies were performed for compounds **4c** and **4e**, as shown in Fig. 9, to explore their binding interactions with human CK2 protein (PDB: 3OWJ) [56]. The performed molecular docking protocol was validated using the docking approach, and the RMSD was found in an acceptable range below 2 Å. The docking study helped to build the initial complex for the MD simulation. Compound **4c** demonstrated a strong interaction with CK2, as evidenced by its lower binding energy of −8.61 kcal mol⁻ [1]. The docking analysis revealed a hydrogen bond between the oxygen atom of the nitro group in **4c** and the NH₂ group of LYS-68 in the CK2 active site. The proximity **4c** of critical residues within the CK2 active site explains its superior biological activity in vitro compared to other derivatives, including **4e**. The binding mode **4e** revealed favourable positioning of the thiadiazole ring and amide group for hydrogen bonding with SER-51. However, the slightly higher binding energy than **4c** suggests that **4e** may have a weaker inhibitory effect on CK2. The molecular docking studies provide valuable insights into the structure–activity relationship of benzimidazole-1,3,4-thiadiazole derivatives. Compound **4c** exhibited the best binding affinity and biological activity, attributed to its favourable interaction with LYS-68 and its strong hydrogen bonding network.

### Molecular dynamics simulation

**Fig. 10:**
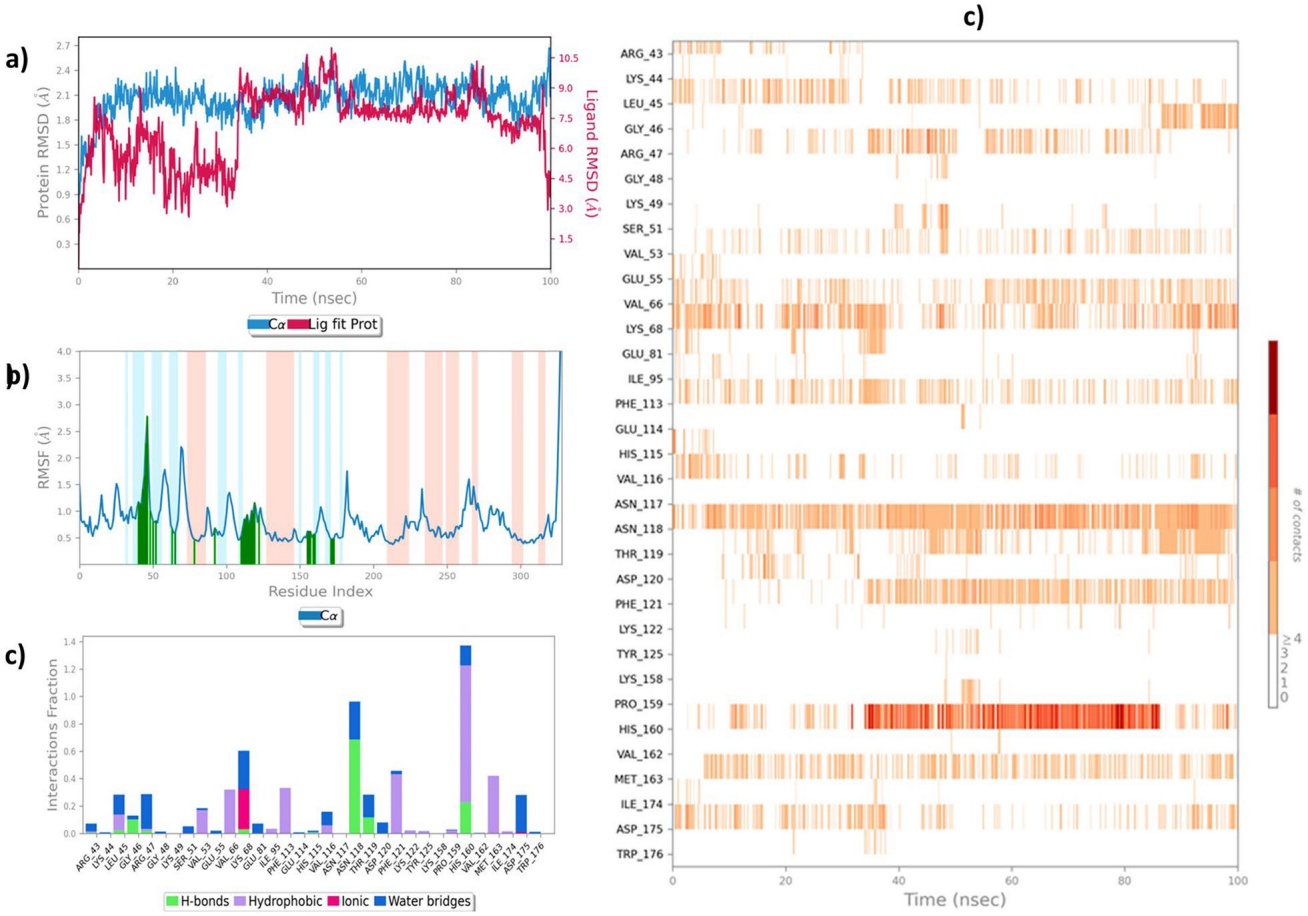
Statistical analysis of MD trajectory; **a**) RMSD profile; **b**) RMSF profile; **c**) Interaction profile; **d**) Consistency of interaction over 100 ns for simulated 4c-CK2 complex

Compound **4c** demonstrated a strong interaction with CK2, as evidenced by its lower binding energy of −8.61 kcal mol⁻ 1 during molecular docking studies. This indicated its potential as a CK2 inhibitor and prompted further investigation into its dynamic behavior under physiological conditions using MD simulation. The MD simulation provided more profound insights into the stability, flexibility, and interaction dynamics of the 4c-CK2 complex. The MD simulation study was performed using Desmond software. The end trajectory was statistically analyzed using a simulation interaction module embedded in Mastero [57]. This approach facilitated the extraction of detailed statistical data, providing quantitative insights into the persistence and strength of interactions between compound **4c** and critical CK2 residues. The use of Desmond and SID ensured a robust and scientifically rigorous evaluation of the molecular behavior and supported the relevance of compound **4c** as a potent CK2 inhibitor. The RMSD analysis was conducted over a 100 ns MD simulation to assess structural stability and ligand binding behavior. The blue trajectory represents the protein backbone RMSD (Cα atoms), while the ligand RMSD is shown in red relative to the protein in Fig. 10a.

The protein exhibited initial fluctuations during the equilibration phase but stabilized after ~ 20 ns. It maintained an average RMSD of approximately 2.0–2.4 Å for the remainder of the simulation. This indicates that the CK2 structure remained stable throughout the simulation without significant conformational changes. The ligand RMSD started with higher fluctuations, indicative of adjustments within the binding pocket. However, the RMSD stabilized after ~ 20 ns. The average RMSD of the ligand ranged between 3 and 10.5 Å. These values suggest that the ligand forms a stable binding pose within the active site of CK2 after an initial equilibration period. The consistent RMSD trajectories for the protein and ligand suggest a well-formed and stable complex. The ligand maintained its interactions within the active site throughout the simulation. The absence of significant deviations in the ligand trajectory supports good binding affinity. This correlates with docking results indicating favorable binding energy (−8.61 kcal/mol). The RMSF was calculated to assess the flexibility of individual residues within the CK2 protein during the 100 ns MD simulation.

The plot in Fig. 10b highlights the fluctuations of the Cα atoms (blue line) across all residues, with peaks indicating regions of higher flexibility. Most residues displayed low RMSF values (below 1.0 Å), indicating overall structural rigidity and stability of the protein throughout the simulation. Notable peaks in RMSF were observed for residues around indices 50, 150, and 300. This suggests localized flexibility in the protein structure. This could correspond to loop regions or solvent-exposed segments of the protein, which are inherently more dynamic than the core regions. Residues in the active site, including those critical for ligand binding (e.g., Lys-68), exhibited relatively low RMSF values. This helps underscore the binding pocket's structural stability in compound **4c**. This observation aligns with the RMSD results, demonstrating a stable protein–ligand complex during the simulation. Highlighted background regions in the plot (Fig. 10b) indicate secondary structure elements (blue for helices, red for sheets). Where lower RMSF values are generally consistent with the rigid nature of these elements. Flexible regions (green bars) correspond to non-structured or loop regions, contributing to the observed peaks.

The interaction fraction chart in Fig. 10c presents the distribution and frequency of different interactions formed by compound **4c** with CK2 protein residues. These interactions include hydrogen bonds (H-bonds), hydrophobic interactions, ionic bonds, and water-mediated bridges. Hydrogen bonding observed with residues such as Thr-118 and Val-160 significantly contributed to the hydrogen bonding. Hydrogen bonds are critical for stabilizing the ligand within the binding pocket and maintaining the specificity of the interaction [58]. Thr-118 exhibited the highest fraction of H-bond interactions. This emphasized its importance in anchoring the ligand. Hydrophobic interactions were prominently observed with residues Val-66, Val-160, and Leu-44. These interactions are essential for enhancing the binding affinity of the ligand's hydrophobic regions with the protein's non-polar regions. Thus, this contributed to the overall stability of the complex. Ionic bonds, though fewer in number, were observed with charged residues such as Lys-68. These electrostatic interactions add specificity and strength to the binding affinity of **4c**.

A substantial fraction of water-mediated interactions were noted, particularly involving residues Glu-81 and Asp-119. Water bridges are auxiliary in stabilizing the ligand–protein complex by forming dynamic networks between the ligand and the protein residues. Lys-68, Thr-118, and Val-160 were particularly prominent in their interaction frequencies. Figure 10d represents the consistency of interactions between the simulated complex of 4c-CK2. This analysis indicated their critical role in the binding mechanism of compound **4c**. This dynamic stability of compound **4c** within the CK2 binding site highlights its potential as a robust inhibitor. The stable binding interactions observed over the simulation period validate its suitability for further experimental and computational studies targeting CK2 inhibition.

### Experimental procedures

#### Synthetic procedure for compounds 4a-m

Compound **3** (1.1 mmol) was dissolved in 1,2-dichloroethane (4 mL) and added to a mixture of compound **2** (0.57 mmol) and 1,2-dichloroethane (5 mL) containing pyridine (1.1 mmol) under nitrogen. The reaction mixture was stirred at room temperature for 6 h. After the completion of reaction, the mixture was evaporated to dryness and diluted with water. Ethyl acetate (3 × 20 mL) was used for extraction, followed by drying of the organic layer with sodium sulfate. The crude product was evaporated and purified by a silica gel column using a mixture of chloroform and methanol (9.5:0.5) as eluent. The pure product was obtained with a yield of 50–70%.

***N-(5-((1H-benzo[d]imidazol-2-yl)methylthio)−1,3,4-thiadiazol-2-yl)−4-fluorobenzamide (4a).*** Off white solid, mp. 205–210 °C. [1]H NMR (DMSO-*d*_*6*_, 400 MHz): *δ* 4.74 (s, 2H), 7.17 (m, 2H, ArH), 7.40 (t, 2H, *J* = 8.8 Hz, ArH), 7.51 (q, 2H, *J* = 3.6 Hz, ArH), 8.17 (dd, *J* = 8.8 Hz & 5.6 Hz, 2H, ArH), 12.66 (br s, 1H, NH), 13.18 (br s, 1H, NH) ppm. [13]C NMR (DMSO-*d*_*6*_, 100 MHz): *δ* 31.21, 115.64, 115.86, 121.86, 127.80, 127.83, 131.31, 131.40, 149.79, 157.99, 160.26, 163.65, 164.24 ppm. IR (KBr): = 3305, 3203, 1672, 653,1535,1508,1431, 1313, 1296, 1232, 1163, 1060, 740 cm^−1^. HRMS (EI) (m/z) calcd for C_17_H_12_FN_5_OS_2_ 385.0467, found 385.0463.

***N-(5-((1H-benzo[d]imidazol-2-yl)methylthio)−1,3,4-thiadiazol-2-yl)−2,4-dichlorobenzamide (4b).*** Off white solid, mp. 195–198 °C. ^1^H NMR (DMSO-*d*_*6*_, 400 MHz): *δ* 4.74 (s, 2H), 7.16 (m, 2H, ArH), 7.51 (q, 2H, *J* = 5.0 Hz, ArH), 7.58 (dd, 1H, *J* = 1.7, 2.1 Hz, ArH), 7.72 (d, 1H, *J* = 8.5 Hz, ArH), 7.80 (d, 1H, *J* = 2.0 Hz, ArH), 12.56 (br s, 1H, NH), 13.36 (br s, 1H, NH) ppm. ^13^C NMR (DMSO-d_6_, 100 MHz): *δ* 31.24, 121.51, 129.47, 130.18, 131.04, 131.68, 132.37, 136.17, 149.79, 158.41, 159.16, 164.21 ppm. IR (KBr): = 3305, 3091, 1676, 1570, 1442, 1429, 1309, 1274, 1105, 1049, 738 cm^−1^. HRMS (EI) (m/z) calcd for C_17_H_11_Cl_2_N_5_OS_2_ 434.9782, found 434.9779.

***N-(5-((1H-benzo[d]imidazol-2-yl)methylthio)−1,3,4-thiadiazol-2-yl)−2-chloro-5-nitrobenzamide (4c).*** Pale brown solid, mp. 200–205 °C. [1]H NMR (DMSO-*d*_*6*_, 400 MHz): *δ* 4.75 (s, 2H), 7.17 (q, 2H, *J* = 3.2 Hz, ArH), 7.51 (q, 2H, *J* = 3.0 Hz, ArH), 7.89 (d, 1H, *J* = 8.8 Hz, ArH), 8.82 (q, 1H, *J* = 2.4 Hz, ArH), 8.62 (d, 1H, *J* = 2.8 Hz, ArH) ppm. [13]C NMR (DMSO-*d*_*6*_, 100 MHz): *δ* 31.13, 121.99, 124.90, 126.75, 131.47, 132.36, 137.42, 145.94, 149.79, 158.57, 159.19, 163.15, 164.90 ppm. IR (KBr): = 3441, 3201, 1674, 1610, 1566, 1525, 1438, 1400, 1346, 1317, 1045, 1049, 738 cm^−1^. HRMS (EI) (m/z) calcd for C_17_H_11_ClN_6_O_3_S_2_ 446.0023, found 446.0014.

***N-(5-((1H-benzo[d]imidazol-2-yl)methylthio)−1,3,4-thiadiazol-2-yl)−2,6-dichloro isonicotinamide (4 d).*** Off white solid, mp. 185–190 °C. ^1^H NMR (DMSO-*d*_*6*_, 400 MHz): *δ* 4.75 (s, 2H), 7.18 (q, 2H, *J* = 3.2 Hz, ArH), 7.53 (q, 2H, *J* = 3.2 Hz, ArH), 8.09 (s, 2H) ppm. ^13^C NMR (DMSO-d_6_, 100 MHz): *δ* 30.99, 115.01, 122.00, 122.29, 122.91, 145.67, 149.74, 149.86, 158.31, 160.84, 162.37 ppm. IR (KBr): = 3439, 3180, 2279, 1581, 1535, 1490, 1440, 1425, 1409, 1357, 1278, 815, 734 cm^−1^. HRMS (EI) (m/z) calcd for C_16_H_10_Cl_2_N_6_OS_2_ 435.9735, found 435.9733.

***N-(5-((1H-benzo[d]imidazol-2-yl)methylthio)−1,3,4-thiadiazol-2-yl)−4-nitrobenzamide (4e).*** Yellow solid, mp. 237–240 °C. ^1^H NMR (DMSO-d_6_, 400 MHz): δ 4.75 (s, 2H), 7.15 (q, 2H, J = 3.2 Hz, ArH), 7.51 (q, 2H, J = 3.2 Hz, ArH), 8.29 (t, 2H, J = 4.4 Hz, ArH), 8.37 (d, 2H, J = 8.8 Hz, ArH), 13.03 (br s, 2H, NH) ppm. ^13^C NMR (DMSO-d_6_, 100 MHz): δ 31.16, 121.9, 123.63, 130.00, 137.26, 149.85, 158.22, 160.48, 164.29 ppm. IR (KBr): = 3415, 3174, 1668, 1604, 1533, 1521, 1492, 1456, 1433, 1400, 1344, 1325, 1097, 752 cm^−1^. HRMS (EI) (m/z) calcd for C_17_H_12_N_6_O_3_S_2_ 412.0412, found 412.0400.

***N-(5-((1H-benzo[d]imidazol-2-yl)methylthio)−1,3,4-thiadiazol-2-yl)−4-chlorobenzamide (4f).*** Off white solid, mp. 225–230 °C. ^1^H NMR (DMSO-*d*_*6*_, 400 MHz): *δ* 4.73 (s, 2H), 7.16 (t, 2H, *J* = 2.8 Hz, ArH), 7.51 (d, 2H, *J* = 3.6 Hz, ArH), 7.63 (d, 2H, *J* = 8.0 Hz, ArH), 8.10 (d, 2H, *J* = 8.4 Hz, ArH), 12.78 (br s, 1H, NH), 13.22 (br s, 1H, NH) ppm. ^13^C NMR (DMSO-d_6_, 100 MHz): *δ* 31.21, 121.87, 128.72, 129.48, 130.14, 131.12, 132.25, 137.98, 149.77, 158.06, 160.23, 164.42 ppm. IR (KBr): = 3263, 1664, 1597, 1552, 1529, 1494, 1433, 1386, 1315, 1298, 1273, 1153, 1105, 1016, 846, 738 cm^−1^. HRMS (EI) (m/z) calcd for C_17_H_12_ClN_5_OS_2_ 401.0172, found 401.0102.

***N*-(5-((1*H*-benzo[d]imidazol-2-yl)methylthio)−1,3,4-thiadiazol-2-yl)−2-chlorobenzamide (4 g).** Pale yellow solid, mp. 195–200 °C. ^1^H NMR (DMSO-d_6_, 400 MHz): δ 4.75 (s, 2H), 7.17 (q, 2H, J = 3.03 Hz, ArH), 7.48 (dt, 1H, J = 1.84, 1.52, 1.84 Hz, ArH), 7.53 (m, 2H, ArH), 7.59 (m, 2H, ArH), 7.67 (dd, 1H, J = 1.0, 1.52 Hz, ArH), 12.57 (br s, 1H, NH), 13.31 (br s, 1H, NH) ppm. ^13^C NMR (DMSO-d_6_, 100 MHz): δ 31.28, 121.88, 127.26, 129.60, 129.85, 130.33, 132.32, 133.50, 149.80, 158.30, 159.16, 165.00 ppm. IR (KBr): = 3448, 3292, 1674, 1591, 1554, 1431, 1311, 1271, 1051, 893, 738 cm^−1^. HRMS (EI) (m/z) calcd for C_17_H_12_ClN_5_OS_2_ 401.0172, found 401.0104.

***N-(5-((1H-benzo[d]imidazol-2-yl)methylthio)−1,3,4-thiadiazol-2-yl) nicotinamide (4 h).*** Off white solid, mp. 175–180 °C. ^1^H NMR (DMSO-*d*_*6*_, 400 MHz): *δ* 5.02 (s, 2H), 7.51 (m, 2H, ArH), 7.61 (q, 1H, *J* = 12.0 Hz, ArH), 7.77 (m, 2H, ArH), 8.43 (m, 1H, ArH), 8.83 (q, 1H, *J* = 6.4 Hz, ArH), 9.20 (d, 1H, *J* = 2.0 Hz, ArH), 13.47 (br s, 1H, NH) ppm. ^13^C NMR (DMSO-*d*_*6*_, 100 MHz): *δ* 31.20,121.87, 143.84, 143.90, 144.62, 144.69, 145.55, 147.71, 148.49, 149.77, 162.77, 165.12 ppm. IR (KBr): = 3655, 3338, 3078, 1886, 1726, 1701, 1670, 1521, 1390, 1313, 1271, 1174, 1153, 1056, 1018, 781 cm^−1^. HRMS (EI) (m/z) calcd for C_16_H_12_N_6_OS_2_ 368.4361, found 368.4350.

***N-(5-((1H-benzo[d]imidazol-2-yl)methylthio)−1,3,4-thiadiazol-2-yl)−4-methoxybenzamide (4i).*** Off white solid, mp. 175–180 °C^. 1^H NMR (DMSO-*d*_*6*_, 400 MHz): *δ* 3.85 (s, 3H), 4.73 (s, 2H), 7.11 (d, 2H, *J* = 8.8 Hz, ArH), 7.17 (q, 2H, *J* = 3.2 Hz, ArH), 7.52 (q, 2H, *J* = 8.8 Hz, ArH), 8.11 (d, 2H, *J* = 8.8 Hz, ArH), 12.97 (br s, 1H, NH) ppm. ^13^C NMR (DMSO-*d*_*6*_, 100 MHz): *δ* 31.22, 55.56, 113.99, 114.71, 121.88, 123.14, 130.55, 149.82, 157.66, 160.36, 163.09, 164.43 ppm. IR (KBr): = 3441, 3296, 1658, 1610, 1550, 1516, 1429, 1317, 1273, 1298, 1259, 1178, 1147, 1026, 837, 738 cm^−1^. HRMS (EI) (m/z) calcd for C_18_H_15_N_5_O_2_S_2_ 397.0067, found 397.0052.

***N-(5-((1H-benzo[d]imidazol-2-yl)methylthio)−1,3,4-thiadiazol-2-yl)−4-methylbenzamide (4j).*** Off white solid, mp. 175–180 °C. ^1^H NMR (DMSO-*d*_*6*_, 400 MHz): *δ* 2.39 (s, 3H), 4.73 (s, 2H), 7.17 (d, 2H, *J* = 2.8 Hz, ArH), 7.36 (d, 2H, *J* = 8.0 Hz, ArH), 7.52 (s, 2H, ArH), 8.0 (d, 2H, *J* = 7.6 Hz, ArH), 12.55 (br s, 1H, NH), 13.06 (br s, 1H, NH) ppm. ^13^C NMR (DMSO-*d*_*6*_, 100 MHz): *δ* 21.10, 31.23, 121.86, 128.37, 128.44, 129.22, 143.52, 149.80, 157.83, 160.24, 165.03 ppm. IR (KBr): = 3441, 3021, 1660, 1614, 1546, 1435, 1301, 1236, 1153, 738 cm^−1^. HRMS (EI) (m/z) calcd for C_18_H_15_N_5_OS_2_ 381.0718, found 381.0700.

***N-(5-((1H-benzo[d]imidazol-2-yl)methylthio)−1,3,4-thiadiazol-2-yl)−3-(trifluoromethyl) benzamide (4 k).*** Pale brown solid, mp. 205–210 °C.^1^H NMR (DMSO-*d*_*6*_, 400 MHz): *δ* 4.76 (s, 2H), 7.18 (q, 2H, *J* = 3.1 Hz, ArH), 7.53 (q, 2H, *J* = 3.1 Hz, ArH), 7.81 (t, 1H, *J* = 7.7 Hz, ArH), 8.04 (d, 1H, *J* = 7.5 Hz, ArH), 8.36 (d, 1H, *J* = 7.5 Hz, ArH), 8.48 (s, 2H), 13.42 (br s, 1H, NH) ppm. ^13^C NMR (DMSO-*d*_*6*_, 100 MHz): *δ* 31.14, 121.92, 125.09, 125.13, 129.18, 129.50, 129.99, 132.39, 132.56, 149.78, 158.18, 160.29, 164.11 ppm. IR (KBr): = 3377, 3169, 1664, 1544, 1438, 1334, 1301, 1246, 1170, 1124, 1074, 742 cm^−1^. HRMS (EI) (m/z) calcd for C_18_H_12_F_3_N_5_OS_2_ 435.0435, found 435.0420.

***N-(5-((1H-benzo[d]imidazol-2-yl)methylthio)−1,3,4-thiadiazol-2-yl)−4-cyanobenzamide (4 l).*** Pale brown solid, mp. 200–205 °C.^1^H NMR (DMSO-*d*_*6*_, 400 MHz): *δ* 4.75 (s, 2H), 7.18 (s, 2H, ArH), 7.53 (s, 2H, ArH), 8.05 (d, 2H, *J* = 7.2 Hz, ArH), 8.22 (d, 1H, *J* = 7.2 Hz, ArH), 13.25 (br s, 1H, NH) ppm. ^13^C NMR (DMSO-*d*_*6*_, 100 MHz): *δ* 31.16, 115.05, 118.07, 121.19, 129.9, 132.59, 132.67, 135.53, 149.76, 158.26, 160.27, 164.38 ppm. IR (KBr): = 3441, 3176, 2229, 1660, 1546, 1404, 1305, 1274, 1056, 1020, 759, 744 cm^−1^. HRMS (EI) (m/z) calcd for C_18_H_12_N_6_OS_2_ 392.0514, found 392.0508.

***N-(5-((1H-benzo[d]imidazol-2-yl)methylthio)−1,3,4-thiadiazol-2-yl)−3,5-dinitrobenzamide (4 m).*** Yellow solid, mp. 175–180 °C. ^1^H NMR (DMSO-*d*_*6*_, 400 MHz): *δ* 4.75 (s, 2H), 7.17 (q, 2H, *J* = 3.1 Hz, ArH), 7.52 (q, 2H, *J* = 3.1 Hz, ArH), 9.01 (s, 1H), 9.23 (s, 2H) ppm. ^13^C NMR (DMSO-*d*_*6*_, 100 MHz): *δ* 30.97, 115.01, 121.84, 122.00, 128.62, 135.44, 148.14, 149.75, 157.99, 163.09 ppm. IR (KBr): = 3311, 3103, 1616, 1541, 1490, 1452, 1413, 1346, 1315, 1235, 719 cm^−1^. HRMS (EI) (m/z) calcd for C_17_H_11_N_7_O_5_S_2_ 457.0263, found 457.0254.

## Conclusion

A new set of amide derivatives containing benzimidazole-1,3,4-thiadiazole were synthesized by introducing various acids with electron-donating and withdrawing substituents to benzimidazole-1,3,4-thiadiazole amine. Amides with electron-withdrawing substituents showed better inhibition on the HeLa cell line compared to those with electron-donating substituents. Among the compounds **4c** and **4e**, electron-withdrawing substituents, showed the best inhibition and had the lowest binding energy with CK2. The docking results provided useful information for designing. The in vitro results and in silico showed a correlation, indicating importance of benzimidazole conjugation with1,3,4-thiadiazole for designing more potent inhibitors for cancer treatment.

## Supplementary Information

Supplementary file 1

## Data Availability

The authors confirm that the data supporting the findings of this study are available within the article and its supplementary materials.
