# Supplementary material for: Novel benzimidazole-1, 3, 4-thiadiazole derivatives as casein kinase-2 inhibitors: synthesis, in vitro and in silico investigations
Source: BMC Chem. 2025 Jun 9;19(1):161. doi: 10.1186/s13065-025-01532-z (PMC12147251; doi:10.1186/s13065-025-01532-z)
Supplement: Supplementary file 1 [file 13065_2025_1532_MOESM1_ESM.docx]

Novel Benzimidazole-1, 3, 4-thiadiazole derivatives as Casein kinase-2 inhibitors: Synthesis, *in vitro* and *in silico* investigations

N. Senthilkumar^1^, S. Sarveswari^1^, Prafulla Choudhari^2^, Somdatta Chaudhari^3^, Imadul Islam^4^, Yasinalli Tamboli^4^*, V. Vijayakumar^1^*

1. Department of Chemistry, Vellore Institute of Technology, Vellore-632 014, Tamil Nadu, India.
2. Department of Pharmaceutical Chemistry, Bharati Vidyapeeth College of Pharmacy, Kolhapur, India
3. Department of Pharmaceutical Chemistry, PES’s Modern College of Pharmacy, Pune 411 044, Maharashtra, India
4. King Abdullah International Medical Research Center (KAIMRC), King Saud Bin Abdulaziz University for Health Sciences, Ministry of National Guard-Health Affairs, Riyadh 14811, Saudi Arabia

Communicative Author:

Dr. V. Vijayakumar

Professor, Department of Chemistry, School of Advanced Sciences,

Vellore Institute of Technology

Vellore-632014, Tamilnadu, India

E-mail: [kvpsvijayakumar@gmail.com](mailto:kvpsvijayakumar@gmail.com)

Mobile Number: +91-9443916746

1. **Chemistry:**

*1.2 Experimental procedures*

*1.2.1 Synthesis of 5-((1H-benzo[d]imidazol-2-yl)methylthio)-1,3,4-thiadiazol-2-amine*

Compound 1 (30 mmol) was added to a suspension of 5-amino-1,3,4-thiadiazole-2-thiol (30 mmol) in 90 mmol of KOH dissolved in 50 mL of water. The mixture was stirred at room temperature for 6 hours. After 6 hours, a pale yellow solid was precipitated in the reaction mixture, and the reaction completion was monitored by TLC. The precipitate was filtered, washed with water, and dried under vacuum to obtain 5-((1*H*-benzo[d]imidazol-2-yl)methylthio)-1,3,4-thiadiazol-2-amine as pale yellow solid, yield 90%; mp 167-168 °C; ^1^H NMR (DMSO-*d*_6_, 400 MHz): *δ* 4.49 (s, 2H), 7.17 (m, 2H), 7.33 (s, 2H), 7.51 (dd, 2H, *J* = 6.8 Hz, *J* = 7.2 Hz), 12.48 (br s, 1H, NH) ppm; ^13^C NMR (DMSO-*d*_6_, 100 MHz): *δ* 32.19, 111.3, 118.61, 121.34, 122.28, 148.68, 150.14, 170.38 ppm. IR (KBr): = 3261, 3140, 2954, 1622, 1523, 1504, 1452, 1435, 1390, 1309, 1273, 1060, 1028, 748 cm-^1^

*1.2.2 General procedure for the synthesis of compound 3.*

Two equivalents of oxalyl chloride were added to one equivalent of acid in 10 mL of dried 1, 2-dichloroethane at 5°C. The resulting suspension was stirred at room temperature for 4 hours. The reaction mixture was checked for conversion of the acid to ester by adding ethanol to a small quantity of it and observing the TLC. Once the conversion was observed, the reaction mixture was evaporated to dryness under a nitrogen atmosphere and used for the next step without further purification.

***1.3 Structural characterization of 4a***

The compounds **4a** was analysed using IR, ^1^H NMR, ^13^C NMR spectroscopy. The IR spectrum displayed two peaks, one in the range of 1672-1676 cm^-1^ due to the presence of a C=O group, and the other in the range of 1653-1658 cm^-1^ due to the presence of a C=N group. The ^1^H NMR spectrum of compound **4a** was chosen as an example. The –CH protons of benzimidazole resonated at δ 7.17 (m) and 7.52 (m) ppm, while the N-H proton of benzimidazole resonated at δ 12.60 ppm. Additionally, the methylene proton at position-2 of benzimidazole resonated at δ 4.74 ppm. Two signals of phenyl protons were observed at δ 7.42 (t) and 8.16 (dd) ppm. The N-H proton of amide resonated at δ 13.18 (s) ppm, indicating the formation of amide. The ^13^C spectrum of **4a** showed 13 signals as expected. In the DEPT-135 spectrum of compound 4a, the -CH carbon of benzimidazole was observed at δ 115.65 and 121.87 ppm, while phenyl ring carbons were observed at δ 131.31 ppm in the positive axis. The methylene carbon attached at position-2 of benzimidazole showed a signal at δ 31.21 ppm in the negative axis. The absence of signals in DEPT-135 at δ 127.80, 149.79, 157.99, 160.26 and 163.65 ppm confirmed these carbons as quaternary carbons. These signals are tentatively assigned to benzimidazole ring carbon, carbon at position-2 of benzimidazole, fluorine-substituted carbon in phenyl ring, and thiadiazole ring. The presence of a signal at δ 164.24 ppm confirmed the carbonyl carbon in the ^13^C NMR. The number of protons and carbons corresponding to compound **4a** are shown in Figure S1.

**Figure S1** Selected ^1^H, ^13^C NMR chemical shift of compound **4a.**

| **Table S1:** Structure, IUPAC name and yield of synthesized compounds **4 (a-m)** | | | | |
| --- | --- | --- | --- | --- |
| Entry | R-COCl  (R) | Products | ICPAC name | Yield (%) |
| 1 |  |  | *N*-(5-((1*H*-benzo[d]imidazol-2-yl)methylthio)-1,3,4-thiadiazol-2-yl)-4-fluorobenzamide | 50 |
| 2 |  |  | *N*-(5-((1*H*-benzo[d]imidazol-2-yl)methylthio)-1,3,4-thiadiazol-2-yl)-2,4-dichlorobenzamide | 65 |
| 3 |  |  | *N*-(5-((1*H*-benzo[d]imidazol-2-yl)methylthio)-1,3,4-thiadiazol-2-yl)-2-chloro-5-nitrobenzamide | 60 |
| 4 |  |  | *N*-(5-((1*H*-benzo[d]imidazol-2-yl)methylthio)-1,3,4-thiadiazol-2-yl)-2,6-dichloroisonicotinamide | 60 |
| 5 |  |  | *N*-(5-((1*H*-benzo[d]imidazol-2-yl)methylthio)-1,3,4-thiadiazol-2-yl)-4-nitrobenzamide | 68 |
| 6 |  |  | *N*-(5-((1*H*-benzo[d]imidazol-2-yl)methylthio)-1,3,4-thiadiazol-2-yl)-4-chlorobenzamide | 70 |
| 7 |  |  | *N*-(5-((1*H*-benzo[d]imidazol-2-yl)methylthio)-1,3,4-thiadiazol-2-yl)-2-chlorobenzamide | 54 |
| 8 |  |  | N-(5-((1*H*-benzo[d] imidazol-2-yl)methyl thio)-1,3,4-thiadiazol-2-yl)nicotinamide | 50 |
| 9 |  |  | *N*-(5-((1*H*-benzo[d] imidazol-2-yl)methyl thio)-1,3,4-thiadiazol-2-yl)-4-methoxy benzamide | 70 |
| 10 |  |  | *N*-(5-((1*H*-benzo[d]imidazol-2-yl)methylthio)-1,3,4-thiadiazol-2-yl)-4-methylbenzamide | 62 |
| 11 |  |  | *N*-(5-((1*H*-benzo[d] imidazol-2-yl)methyl thio)-1,3,4-thiadiazol-2-yl)-3-(trifluoromethyl) benzamide | 66 |
| 12 |  |  | *N*-(5-((1*H*-benzo[d] imidazol-2-yl) methylthio)-1,3,4-thiadiazol-2-yl)-4-cyanobenzamide | 53 |
| 13 |  |  | *N*-(5-((1*H*-benzo[d] imidazol-2-yl) methylthio)-1,3,4-thiadiazol-2-yl)-3,5-dinitrobenzamide | 57 |

1. ***In silico studies***

***2.1 Density functional theory (DFT) study***

The chemical reactivity descriptors of benzimidazole-1,3,4-thiadiazole derivatives (**4a**–**4m**) were calculated using the DFT method in ORCA. This combination of DFT study, ADMET, and physicochemical screening provided a comprehensive understanding of their potential as drug candidates. The DFT study helped to estimate the chemical reactivity descriptors and frontier molecular orbitals for the synthesized compounds.^1^ The HOMO and LUMO energy levels are key indicators for the ability of a compound to donate and accept electrons. The HOMO-LUMO gap (HLG) of **4c** (3.459 eV) is more significant than that of **4m** (3.016 eV), indicating that, according to DFT predictions, **4m** should be more chemically reactive. However, the higher reactivity of **4m** does not translate to greater *in vitro* activity. This suggests that other factors may play a more significant role in determining its biological efficacy.^2^ These factors could include the ability of compounds to bind to the target or its stability in a biological environment.^3^

**Table S2**. Energies of chemical reactivity descriptors for synthesized benzimidazole-1, 3, 4-thiadiazole derivatives (**4a**–**4m**) calculated using the DFT method.

| **Code** | **HOMO (eV)** | **LUMO (eV)** | **HLG (eV)** | **DM (Debye)** | **IP (eV)** | **EA (eV)** | **χ (eV)** | **µ (eV)** | **η (eV)** | **ω (eV)** |
| --- | --- | --- | --- | --- | --- | --- | --- | --- | --- | --- |
| **4a** | -6.367 | -1.698 | 4.669 | 4.265 | 6.367 | 1.698 | 4.033 | -4.033 | 2.335 | 3.483 |
| **4b** | -6.375 | -1.708 | 4.667 | 3.469 | 6.375 | 1.708 | 4.042 | -4.042 | 2.334 | 3.500 |
| **4c** | -6.439 | -2.980 | 3.459 | 7.661 | 6.439 | 2.980 | 4.710 | -4.710 | 1.730 | 6.412 |
| **4d** | -6.433 | -2.294 | 4.139 | 5.861 | 6.433 | 2.294 | 4.364 | -4.364 | 2.070 | 4.600 |
| **4e** | -6.444 | -2.957 | 3.487 | 7.073 | 6.444 | 2.957 | 4.701 | -4.701 | 1.744 | 6.336 |
| **4f** | -6.378 | -1.754 | 4.624 | 4.103 | 6.378 | 1.754 | 4.066 | -4.066 | 2.312 | 3.575 |
| **4g** | -6.349 | -1.600 | 4.749 | 2.942 | 6.349 | 1.600 | 3.975 | -3.975 | 2.375 | 3.326 |
| **4h** | -6.396 | -1.901 | 4.495 | 6.196 | 6.396 | 1.901 | 4.149 | -4.149 | 2.248 | 3.829 |
| **4i** | -6.184 | -1.529 | 4.655 | 2.866 | 6.184 | 1.529 | 3.857 | -3.857 | 2.328 | 3.195 |
| **4j** | -6.327 | -1.577 | 4.750 | 3.983 | 6.327 | 1.577 | 3.952 | -3.952 | 2.375 | 3.288 |
| **4k** | -6.374 | -1.923 | 4.451 | 5.359 | 6.374 | 1.923 | 4.149 | -4.149 | 2.226 | 3.867 |
| **4l** | -6.434 | -2.232 | 4.202 | 6.865 | 6.434 | 2.232 | 4.333 | -4.333 | 2.101 | 4.468 |
| **4m** | -6.489 | -3.473 | 3.016 | 7.436 | 6.489 | 3.473 | 4.981 | -4.981 | 1.508 | 8.226 |

According to this order, **4m**, with the smallest HOMO-LUMO gap, should theoretically exhibit the highest reactivity, followed by **4c**. However, *in vitro* studies show that **4c** exhibits superior biological activity to **4m**. This discrepancy may arise due to the importance of other molecular properties, such as solubility, bioavailability, and target specificity, which influence the actual bioactivity in a cellular environment. Compound **4c** has a slightly higher HOMO-LUMO gap. However, it might interact more effectively with the biological target. This suggests its molecular structure may confer enhanced binding affinity or stability. These effects are not fully captured by the *in silico* reactivity models. The DFT results provide essential insights into the electronic structure of these compounds. However, these results must be interpreted in conjunction with experimental findings. For instance, the dipole moment (DM) of **4c** (7.661 D) is significantly higher than that of **4m** (3.016 D). This indicates that **4c** may exhibit stronger intermolecular interactions in polar environments, potentially leading to better solubility and interactions with biological targets. The higher DM of **4c** could also enhance its ability to interact with proteins or nucleic acids, which is critical for its activity in biological systems.

Additionally, **4c** demonstrated the highest DM among the derivatives, which may correlate with its favorable pharmacokinetic properties. Higher DM generally suggests better solubility and permeability in polar environments, potentially explaining enhanced *in vitro* activity of **4c**. Compound **4c** exhibited intestinal absorption of 89.062% and favourable distribution characteristics with a low BBB permeability (log BB -1.592). Its log Papp value of 1.114 further supports its potential for good oral bioavailability. Despite its relatively high DM, **4c** showed favourable absorption and permeability, indicating that these physicochemical properties contribute to enhanced *in vitro* activity. The discrepancy between the reactivity order and experimental results underscores the importance of considering multiple factors- such as molecular interactions, stability, and solubility - when predicting the biological activity of compound **4c**.

***2.2 Molecular docking study***

Molecular docking studies were performed for compounds **4c** and **4e** to explore their binding interactions with human CK2 protein (PDB: 3OWJ).^4^ The docking results provided insights into the molecular basis of their biological activity, correlating with the observed *in vitro* data. This interaction occurred at a distance of 3.5 Å, contributing significantly to stabilizing the ligand-protein complex. The binding mode of **4c** within the CK2 cavity was characterized by favourable positioning of the nitro group for interaction, enhancing its inhibitory potential.

As illustrated in **Figure 5**, the binding mode shows the ligand tightly nestled in the binding pocket, supported by hydrogen bonding networks and van der Waals interactions. This high affinity is consistent with the ADMET profile of **4c**, indicating favourable pharmacokinetic properties such as high absorption and efficient distribution. Compound **4e** also exhibited significant binding affinity with CK2, with a binding energy of -7.93 kcal mol⁻¹, slightly weaker than **4c**. Docking results highlighted two key interactions: a hydrogen bond between the hydrogen atom of the amide NH group in **4e** and the OH group of SER-51 at a distance of 2.5 Å. Another hydrogen bond between the nitrogen atom of the thiadiazole ring in **4e** and the OH group of SER-51 at a distance of 3.1 Å. These dual interactions contributed to stabilizing the ligand in the CK2 binding pocket.

This correlates with the ADMET profile, also showed slightly lower absorption and distribution metrics for **4e** than **4c**. The docking studies for **4c** and **4e** helped explain their biological activity by elucidating key molecular interactions within the CK2 binding pocket. Compound **4c** demonstrated the most vital binding energy and specific interaction with LYS-68 and was also the most active compound in *in vitro* studies. This suggests that the formation of a stable ligand-protein complex is critical for biological activity. Conversely, compound **4e**, despite forming multiple interactions with SER-51, exhibited slightly weaker binding energy and lower *in vitro* activity. This emphasizes the role of interaction specificity and ligand orientation in determining biological outcomes. The dual hydrogen bonding of **4e** could not compensate for the absence of stronger interactions such as those observed for **4c**. These findings correlate well with its ADMET properties, which indicate excellent absorption, distribution, and solubility. Compound **4e**, although active, demonstrated slightly weaker interactions, which may explain its comparatively lower activity. This study underscores the importance of docking analyses in understanding ligand-protein interactions. This can guide the optimization of drug candidates for enhanced efficacy when combined with ADMET and physicochemical properties.

***2.3 Molecular dynamics simulation***

The MD simulation provided more profound insights into the stability, flexibility, and interaction dynamics of the 4c-CK2 complex. The MD simulation study was performed using Desmond software. The end trajectory was statistically analyzed using a simulation interaction module embedded in Mastero.^5^ This approach facilitated the extraction of detailed statistical data, providing quantitative insights into the persistence and strength of interactions between compound **4c** and critical CK2 residues. The use of Desmond and SID ensured a robust and scientifically rigorous evaluation of the molecular behaviour and supported the relevance of compound **4c** as a potent CK2 inhibitor.

The blue trajectory represents the protein backbone RMSD (Cα atoms), while the ligand RMSD is shown in red relative to the protein in **Figure 5a**. The ligand RMSD started with higher fluctuations, indicative of adjustments within the binding pocket. However, the RMSD stabilized after ~20 ns. The average RMSD of the ligand ranged between 3–10.5 Å. These values suggest that the ligand forms a stable binding pose within the active site of CK2 after an initial equilibration period. The consistent RMSD trajectories for the protein and ligand suggest a well-formed and stable complex. The ligand maintained its interactions within the active site throughout the simulation. The absence of significant deviations in the ligand trajectory supports good binding affinity.^6^ This correlates with docking results indicating favorable binding energy (-8.61 kcal/mol).

Notable peaks in RMSF were observed for residues around indices 50, 150, and 300. This suggests localized flexibility in the protein structure. This could correspond to loop regions or solvent-exposed segments of the protein, which are inherently more dynamic than the core regions. Residues in the active site, including those critical for ligand binding (e.g., Lys-68), exhibited relatively low RMSF values. This helps to underscore the structural stability of the binding pocket in the presence of compound **4c**. Highlighted background regions in the plot (**Figure 5b**) indicate secondary structure elements (blue for helices, red for sheets) where lower RMSF values are generally consistent with the rigid nature of these elements. Flexible regions (green bars) correspond to non-structured or loop regions, which contributed to the observed peaks.

Hydrogen bonds are critical for stabilizing the ligand within the binding pocket and maintaining the specificity of the interaction. Thr-118 exhibited the highest fraction of H-bond interactions. This emphasized its importance in anchoring the ligand. Hydrophobic interactions were prominently observed with residues Val-66, Val-160, and Leu-44. These interactions are essential for enhancing the binding affinity of hydrophobic regions of the ligand with the non-polar regions of the protein. Thus, this contributed to the overall stability of the complex. Ionic bonds, though fewer in number, were observed with charged residues such as Lys-68. These electrostatic interactions add specificity and strength to the binding affinity of **4c**. A substantial fraction of water-mediated interactions were noted, particularly involving residues Glu-81 and Asp-119. Water bridges are auxiliary in stabilizing the ligand-protein complex by forming dynamic networks between the ligand and the protein residues. Lys-68, Thr-118, and Val-160 were particularly prominent in their interaction frequencies. This analysis indicated their critical role in the binding mechanism of compound **4c**. This dynamic stability of compound **4c** within the CK2 binding site highlights its potential as a robust inhibitor.

***2.4 In silico druglikeness and ADMET assessment***

The drug-likeness and physicochemical properties of benzimidazole-1,3,4-thiadiazole derivatives (**4a**–**4m**) were evaluated using SwissADME.^7^ The predicted values are discussed in **Table S3**. The molecular weight (MW) of the compounds ranged from 368.44 to 446.89 g/mol. The MW remained within the acceptable threshold for drug-likeness according to Lipinski’s Rule of Five. The calculated lipophilicity (XLogP) values varied from 2.4 to 4.73. These values indicated moderate hydrophobicity suitable for oral delivery. The hydrogen bond acceptors (HBA) ranged between 4 and 6, while the hydrogen bond donors (HBD) were consistently 2 across all compounds. This balance supports favourable solubility and permeability profiles.

**Table S3.** Predicted physicochemical properties and drug-likeness profile of benzimidazole-1, 3, 4-thiadiazole derivatives (**4a**–**4m**).

| **Entry** | **MW (g/mol)** | **xLogP** | **HBA** | **HBD** | **MR** | **TPSA** | **nRot** | **Lipinski's Rule (Ro5)** | **Ghose's Rule** | **Veber's Rule** | **Egan's Rule** | **Muegge's Rule** |
| --- | --- | --- | --- | --- | --- | --- | --- | --- | --- | --- | --- | --- |
| **4a** | 385.44 | 3.57 | 5 | 2 | 99.94 | 137.1 | 6 | + | + | + | - | + |
| **4b** | 436.34 | 4.73 | 4 | 2 | 110 | 137.1 | 6 | + | + | + | - | + |
| **4c** | 446.89 | 3.93 | 6 | 2 | 113.81 | 182.92 | 7 | + | + | - | - | - |
| **4d** | 437.33 | 4.33 | 5 | 2 | 107.8 | 149.99 | 6 | + | + | - | - | + |
| **4e** | 412.45 | 3.3 | 6 | 2 | 108.8 | 182.92 | 7 | + | + | - | - | - |
| **4f** | 401.89 | 4.1 | 4 | 2 | 104.99 | 137.1 | 6 | + | + | + | - | + |
| **4g** | 401.89 | 4.1 | 4 | 2 | 104.99 | 137.1 | 6 | + | + | + | - | + |
| **4h** | 368.44 | 2.4 | 5 | 2 | 97.78 | 149.99 | 6 | + | + | - | - | + |
| **4i** | 397.47 | 3.45 | 5 | 2 | 106.47 | 146.33 | 7 | + | + | - | - | + |
| **4j** | 381.47 | 3.84 | 4 | 2 | 104.95 | 137.1 | 6 | + | + | + | - | + |
| **4k** | 435.45 | 4.36 | 7 | 2 | 104.98 | 137.1 | 7 | + | - | + | - | + |
| **4l** | 392.46 | 3.19 | 5 | 2 | 104.7 | 160.89 | 6 | + | + | - | - | - |
| **4m** | 385.44 | 3.57 | 5 | 2 | 99.94 | 137.1 | 6 | + | + | + | - | + |

Topological polar surface area (TPSA) values spanned from 104.95 to 182.92 Å², with compounds like **4c** and **4e** exceeding 140 Å², suggesting potential limitations in membrane permeability for these derivatives. The number of rotatable bonds (nRot) was within 6 to 7 for all compounds. The molar refractivity (MR) values ranged between 97.78 and 113.81, supporting structural diversity among the derivatives. Compliance with various drug-likeness rules was assessed. All compounds adhered to Lipinski's Rule of Five, Ghose's Rule, and Veber's Rule, signifying favourable physicochemical properties for oral bioavailability. However, some compounds failed to comply with Egan's and Muegge's rules, primarily due to high TPSA or XLogP values.

For instance, compounds **4c** and **4e**, with TPSA above 140 Å², might face challenges in passive permeability. This analysis highlights the promising drug-likeness profiles of these derivatives. Most compounds meet key drug-likeness criteria, showing potential as lead candidates for further study. The pharmacokinetic and toxicity profiles of benzimidazole-1,3,4-thiadiazole derivatives (**4a**–**4m**) were evaluated using pkCSM. The predicted values are discussed in **Table S4**.

**Table S4.** Predicted pharmacokinetic (ADMET) profile of benzimidazole-1, 3, 4-thiadiazole derivatives (**4a**–**4m**).

| **Entry** | **Absorption** | | **Distribution** | | | **Metabolism** | | | | | | | **Excretion** | **Toxicity** |
| --- | --- | --- | --- | --- | --- | --- | --- | --- | --- | --- | --- | --- | --- | --- |
|  | **Intestinal absorption (human)** | **Caco-2** | **VDss (human)** | **BBB permeability** | **CNS permeability** | **Substrate** | | **Inhibitors** | | | | | **Total clearance** | **AIMS toxicity** |
|  |  |  |  |  |  | **CYP** | | | | | | |  |  |
|  |  |  |  |  |  | **2D6** | **3A4** | **1A2** | **2C19** | **2C9** | **2D6** | **3A4** |  |  |
|  | **Numeric (%absorbed)** | **Numeric (log Papp)** | **Numeric (log L kg^-1^)** | **Numeric (log BB)** | **Numeric (log PS)** | **Categorical (Yes/No)** | | | | | | | **Numeric (log mL min ^-1^ kg ^-1^)** | **Categorical (Yes/No)** |
| **4a** | 80.686 | 1.114 | -0.115 | -1.365 | -2.467 | No | Yes | Yes | Yes | Yes | No | Yes | 0.666 | Yes |
| **4b** | 78.461 | 1.151 | -0.035 | -1.559 | -2.129 | No | No | Yes | Yes | Yes | No | Yes | 0.591 | Yes |
| **4c** | 89.062 | -0.153 | 0.09 | -1.592 | -2.485 | No | Yes | Yes | Yes | Yes | No | Yes | 0.428 | Yes |
| **4d** | 90.844 | 0.402 | -0.196 | -1.799 | -2.459 | No | No | Yes | Yes | Yes | No | Yes | 0.349 | Yes |
| **4e** | 87.811 | -0.104 | 0.093 | -1.426 | -2.591 | No | Yes | Yes | Yes | Yes | No | Yes | 0.472 | Yes |
| **4f** | 79.682 | 1.154 | -0.016 | -1.386 | -2.242 | No | No | Yes | Yes | Yes | No | No | 0.705 | Yes |
| **4g** | 80.31 | 1.154 | -0.012 | -1.379 | -2.244 | No | No | Yes | Yes | Yes | No | Yes | 0.701 | Yes |
| **4h** | 94.412 | 0.834 | -0.218 | -1.422 | -2.701 | No | No | Yes | Yes | Yes | Yes | Yes | 0.731 | Yes |
| **4i** | 89.527 | 0.439 | -0.113 | -1.355 | -2.596 | No | No | Yes | Yes | Yes | No | Yes | 0.735 | Yes |
| **4j** | 81.14 | 1.164 | -0.004 | -1.233 | -2.283 | No | No | Yes | Yes | Yes | No | Yes | 0.717 | Yes |
| **4k** | 78.708 | 1.17 | -0.035 | -1.5 | -2.205 | No | No | Yes | Yes | Yes | No | Yes | 0.519 | Yes |
| **4l** | 88.667 | 0.335 | -0.014 | -1.271 | -2.421 | No | No | Yes | Yes | Yes | No | Yes | 0.733 | Yes |
| **4m** | 84.613 | -0.441 | 0.175 | -1.646 | -2.856 | No | Yes | Yes | Yes | Yes | No | Yes | 0.178 | Yes |

The intestinal absorption (% absorbed) ranged between 78.46% and 94.41%, indicating high absorption potential for all compounds. Caco-2 permeability values (log Papp) varied slightly, with most compounds exceeding 1.0, supporting good membrane permeability. However, compound **4m** displayed the lowest permeability (-0.441), suggesting limited absorption efficiency. For distribution, the steady-state volume of distribution (log VDss) ranged from -0.218 to 0.175, signifying moderate tissue distribution. The blood-brain barrier (BBB) permeability (log BB) values for all derivatives were below -1.0. This suggests that these compounds are unlikely to cross the BBB. Consequently, the risk of side effects on the central nervous system (CNS) is minimal. Similarly, CNS permeability (log PS) values were well below the threshold, confirming low penetration into the CNS. Metabolism profiling showed that most compounds were potential substrates for cytochrome P450 (CYP) enzymes, such as CYP2D6 and CYP3A4. All derivatives were predicted to inhibit multiple CYP isoforms (CYP1A2, CYP2C19, and CYP3A4).

This suggests possible drug-drug interaction risks. Notably, compounds **4a** and **4b** were not substrates for CYP3A4, which might provide an advantage in reducing first-pass metabolism. The total clearance values ranged from 0.178 to 0.735 log mL/min/kg. This indicates moderate clearance rates. Compound **4m** exhibited the lowest clearance, potentially leading to prolonged systemic exposure. Toxicity prediction revealed that all compounds showed a positive AMES test, indicating potential mutagenicity concerns. These results highlight favourable ADMET profiles for most derivatives, with notable absorption and distribution characteristics. However, the risk of CYP enzyme inhibition and potential mutagenicity warrants further investigation and optimization to improve their safety profiles. This analysis underscores the importance of balancing pharmacokinetic and toxicity parameters during drug development.

**Materials and methods**

***Materials***

Analytical thin-layer chromatography (TLC) was performed on precoated silica gel 60 F254 plates (Merck). Visualization was performed by ultraviolet light and staining with Iodine, ninhydrin and potassium permanganate. Organic Purification techniques were performed by column chromatography using 60-120 mesh silica gel. Proton nuclear magnetic resonance (1H NMR) and 13C NMR spectra were acquired using Bruker 400 MHz spectrometers. Chemical shifts were reported in parts per million (ppm) and were calibrated to residual solvent peaks: proton (CDCl3 7.26 ppm & DMSO-d6 2.50 ppm) for 1H NMR and carbon (CDCl3 77.0 ppm & DMSO-d6 39.52 ppm) for 13C NMR. For 1H NMR, coupling constants (J) were reported in Hz. Multiplicities were reported using the following abbreviations: s = singlet; br s = broad singlet; d = doublet; dd = doublet of doublet; t = triplet; q = quartet; m = multiplet. Infrared spectroscopic data was recorded with the KBr pellet plates using SHIMADZU Infrared spectrophotometer. High-resolution mass spectra were recorded on a Mass-EI-Quadrupole spectrometer. Cytotoxicity study was visualized by Lobomed trinocular inverted microscope TCM 400.

***Ligand structure preparation***

The chemical structures of synthesized benzimidazole-1, 3, 4-thiadiazole derivatives were sketched in ChemDraw 23.1.1 and subsequently protonated with BIOVIA Discovery Studio.^8^ Energy minimization of the ligand structures was performed in PyRx 0.8 through the Open Babel module. MMFF94 force field and optimizing the structures using the steepest descent method were applied for energy minimization.^9^

***In silico druglikeness and ADMET assessment***

The drug-likeness and ADMET properties of the synthesized benzimidazole-1,3,4-thiadiazole derivatives were assessed using the SwissADME and pkCSM web servers.^10,11^

***Density functional theory (DFT) study***

DFT calculations were performed to evaluate the frontier molecular orbital (FMO) energies and reactivity descriptors of benzimidazole-1,3,4-thiadiazole derivatives. The calculations utilized ORCA 5.0.4 with the B3LYP functional and def2-TZVP basis set.^12^ Input files were prepared, and outputs were analyzed using Avogadro.^13^ Reactivity descriptors for the screened compounds were calculated using equations of Koopmans' theory.^14,15^

***Molecular docking study***

The 3-dimensional structures of CK2 were obtained from the Protein Data Bank (PDB).^16^ Co-crystallized ligands were identified and removed from the target proteins, and then crystallographic water molecules were eliminated from the 3D coordinate file. The structures of the synthesized compounds were created using ChemSketch and then converted using Corina. Automated docking studies were performed using Auto-Dock version 4.0. The 3D structure of HPK-CK2 (PDB code: 3OWJ) was implemented through the graphical user interface AutoDockTools (ADT 1.4.6).^17,18^ ADT 1.4.6 was also used to set up the enzyme by adding all hydrogens, loading Kollman United Atoms charges, and merging non-polar hydrogens to carbon atoms. Generated PDB qt files were saved. The 3D structures of ligand molecules were constructed and optimized, and AUTODOCK 4.0 was used for all docking calculations. The AutoDockTools interface was also used to generate the grid and docking parameter files and calculate docking scores for β-lactam antibiotics and penicillin derivatives. Finally, protein-ligand complexes were analyzed and visualized using the PyMOL viewer.

***Molecular dynamics simulation***

Molecular dynamics (MD) simulations were carried out using the Desmond module within the Schrödinger Suite to analyze the protein-ligand behavior under a dynamic atmosphere.^19-21^ The simulation systems were constructed using the Desmond System Builder in Maestro. This placed the complex in an orthorhombic box, solvated with the SPC water model [15].^22^ This system was neutralized by adding 0.15 M NaCl to simulate physiological conditions.^23-25^ Prior to the MD simulations, the system underwent energy minimization with the OPLS2005 force field to remove steric clashes and optimize the initial structure. The equilibration process involved two steps: first, a 10 ns NVT ensemble to stabilize the temperature of the system, followed by a 12 ns NPT ensemble to equilibrate the system under constant pressure.^26^ The temperature was controlled at 310.15 K using the Nose-Hoover chain thermostat (1.0 ps relaxation time).^27^ The pressure was maintained at 1 bar with the Martyna-Tuckerman-Klein barostat (2 ps relaxation time). The final production run was performed for 100 ns, with periodic frame collection every 50 ps to generate 1000 snapshots. The resulting trajectories were analyzed using statistical methods to assess the stability and dynamic behavior of the complex over the simulation period.

***Cytotoxicity assay***

Cytotoxicity studies of the compounds were carried out on human cervical cancer cells (HeLa) which was obtained from National Centre for Cell Science, Pune, India. Cell viability was carried out using the MTT assay method. The HeLa was grown in Eagles minimum essential medium containing 10% fetal bovine serum (FBS). For the screening experiment, the cells were seeded into 96-well plates in 100 *μ*L of the respective medium containing 10% FBS, at a plating density of 10000 cells/well, and incubated at 37 °C, under conditions of 5% CO_2_, 95% air, and 100% relative humidity for 24h prior to the addition of compounds. The compounds were dissolved in DMSO and diluted in the respective medium containing 1% FBS. After 24 h, the medium was replaced with the respective medium with 1% FBS containing the compounds at various concentrations and incubated at 37°C under conditions of 5% CO2, 95% air, and 100% relative humidity for 48 h. Triplication was maintained, and the medium not containing the compounds served as the control. After 48 h, 10*μ*L of MTT (5 mg/mL) in phosphate buffered saline (PBS) was added to each well and incubated at 37 °C for 4 h. The medium with MTT was then flicked off, and the formed formazan crystals were dissolved in 100 *μ*L of DMSO. The absorbance was then measured at 570 nm using a microplate reader. The percentage of cell inhibition was determined using the following formula, and a graph was plotted with the percentage of cell inhibition versus concentration.^28^

% cell inhibition was determined using the following formula. IC_50_ value was calculated using a graph plot between % cell inhibition and concentration.

% Cell Inhibition = 100- Abs (sample)/Abs (control) x100


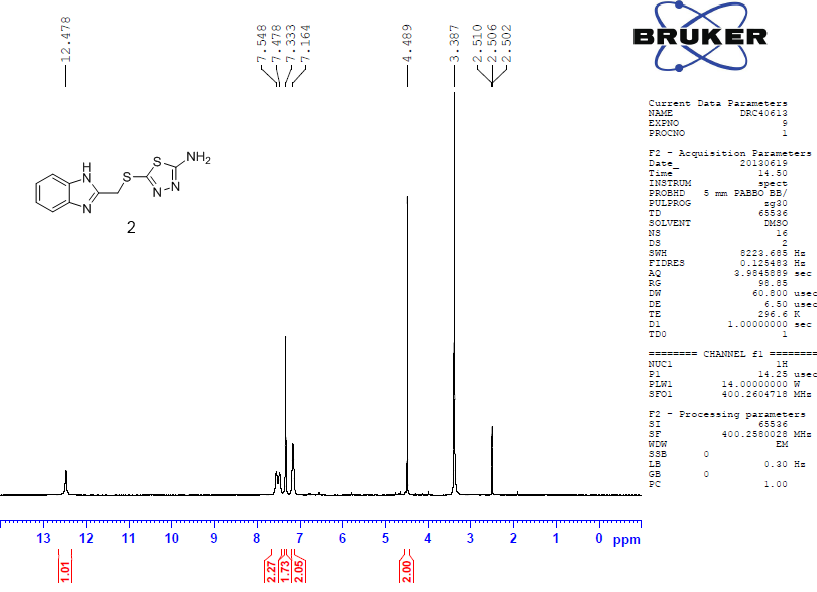

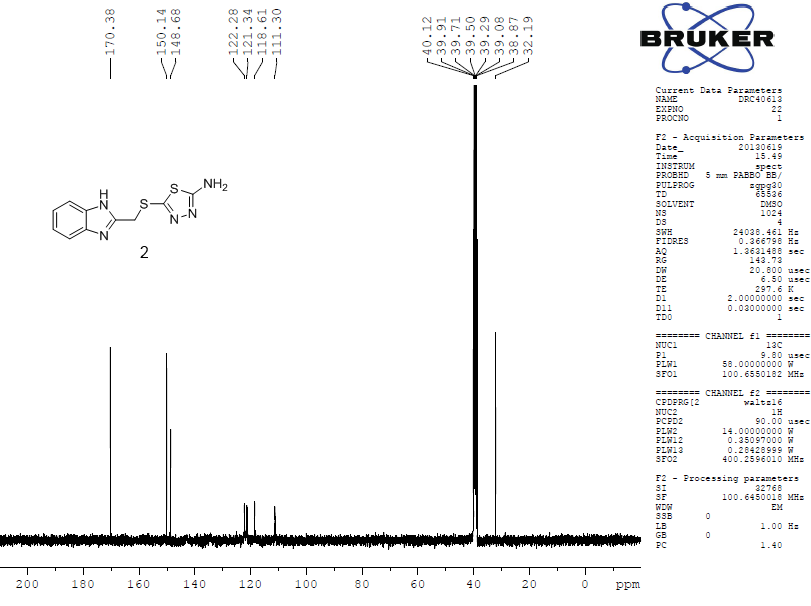

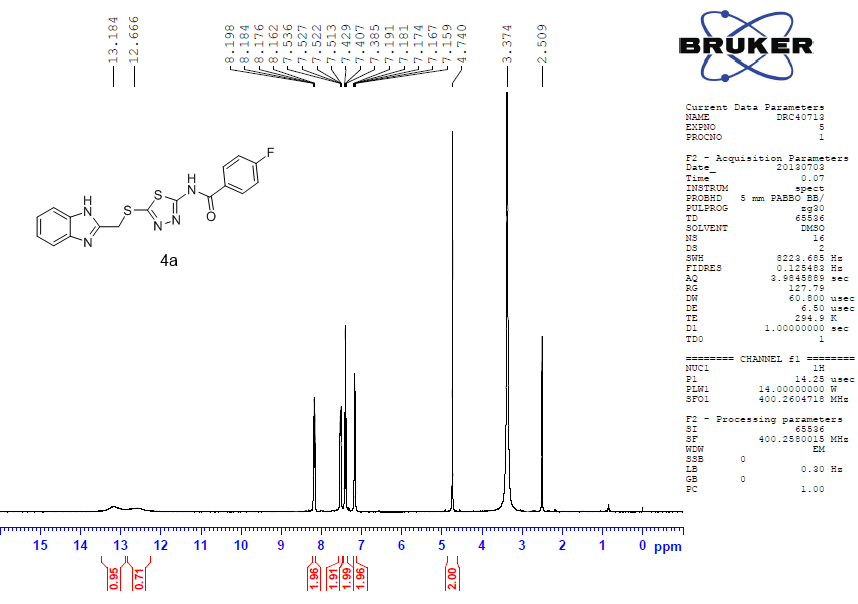

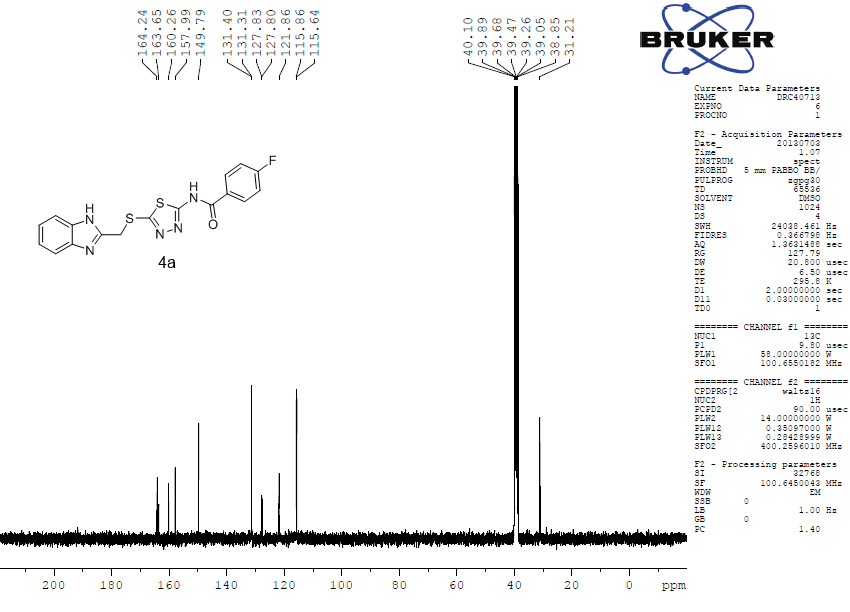

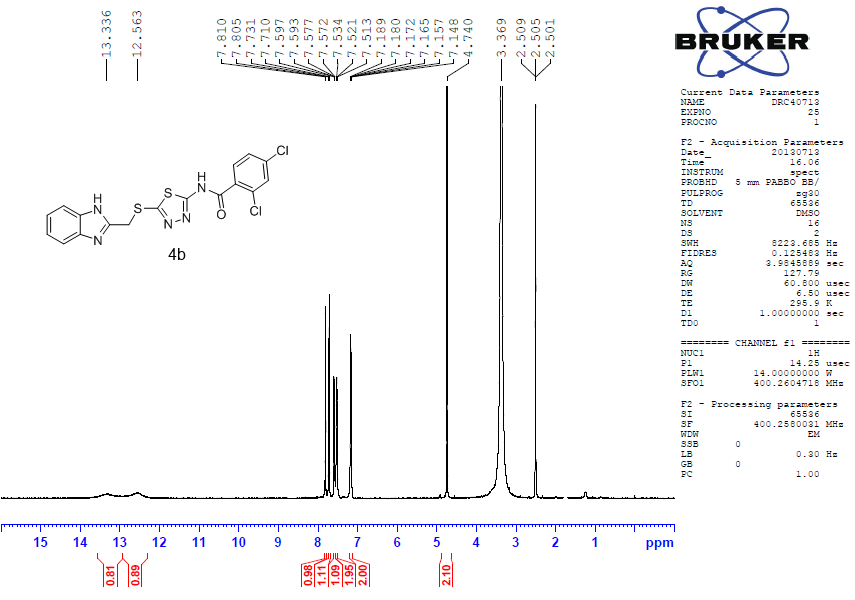

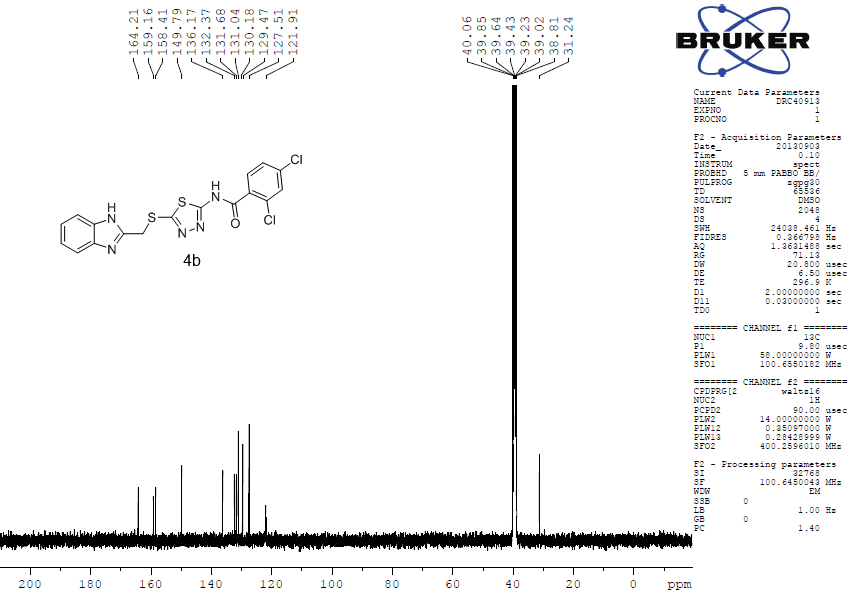

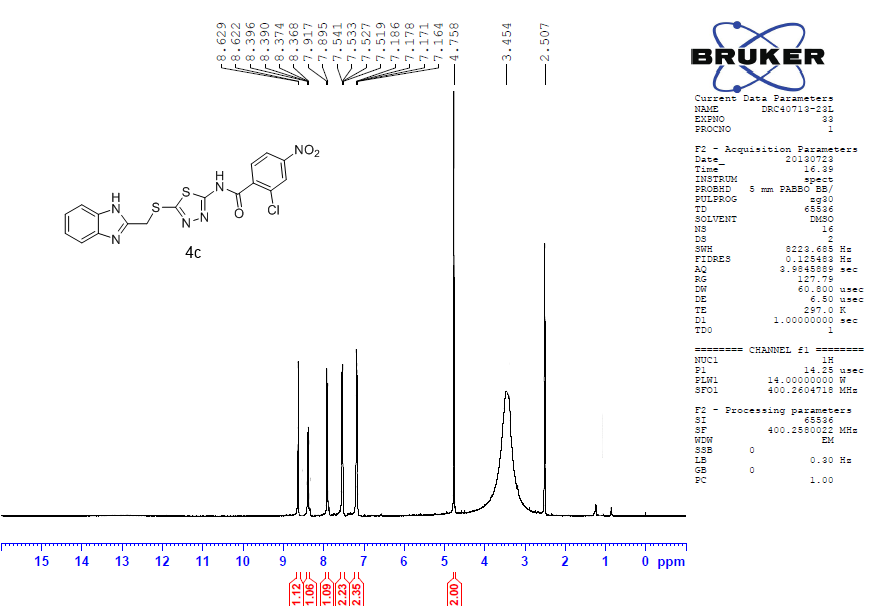

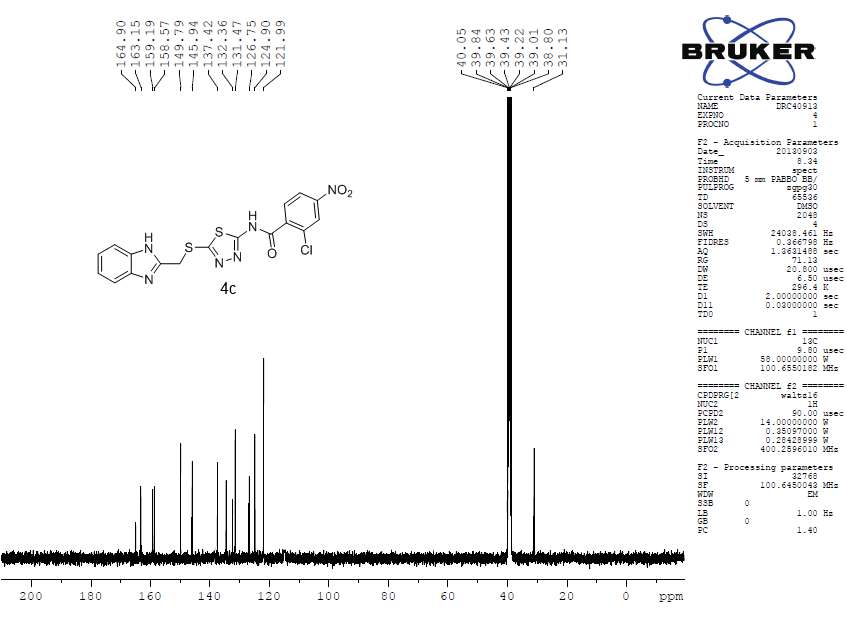

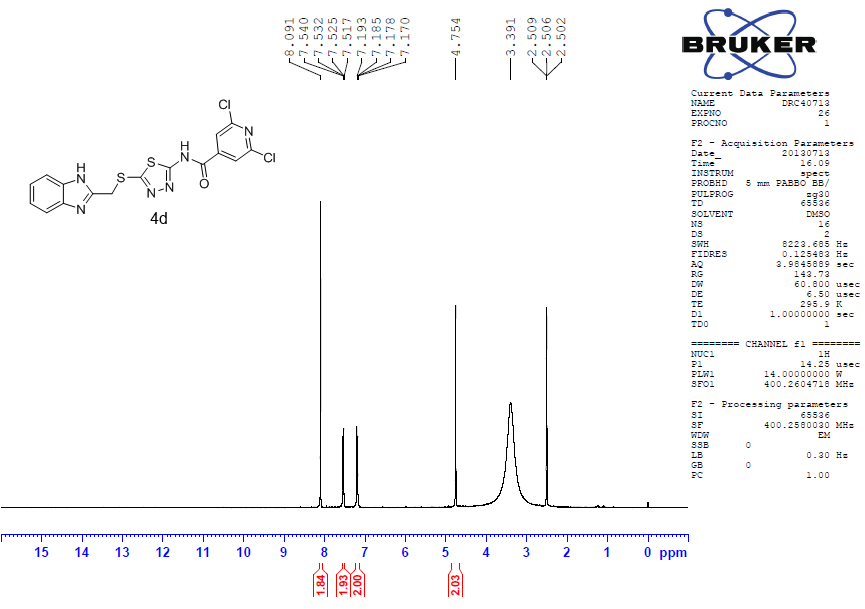

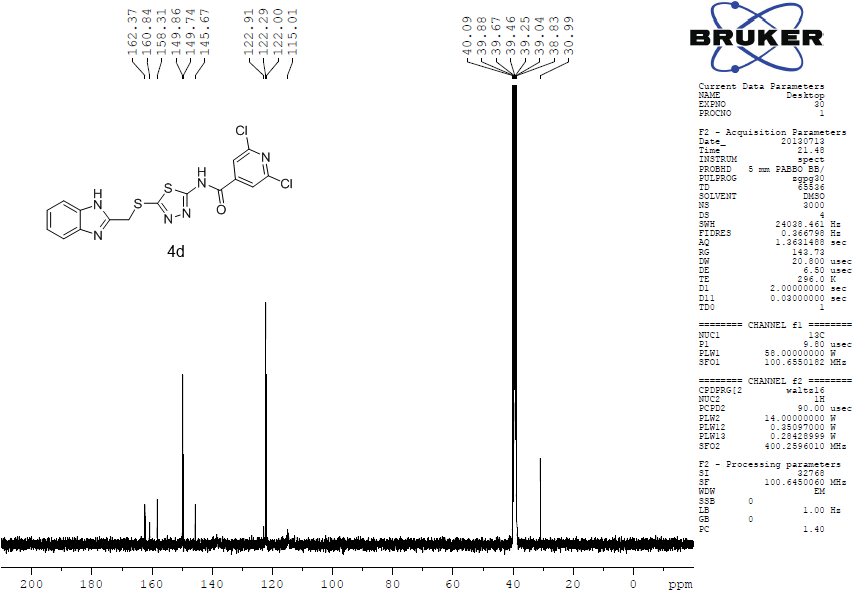

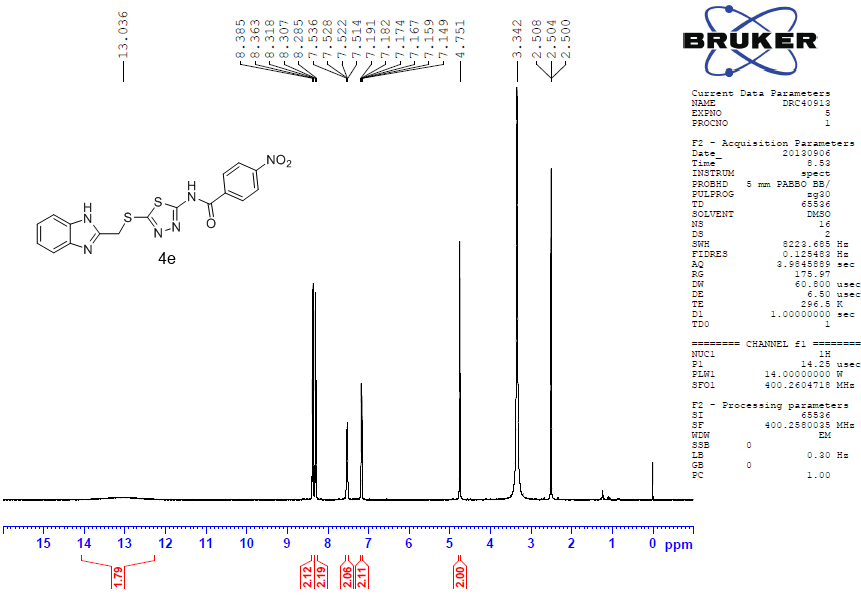

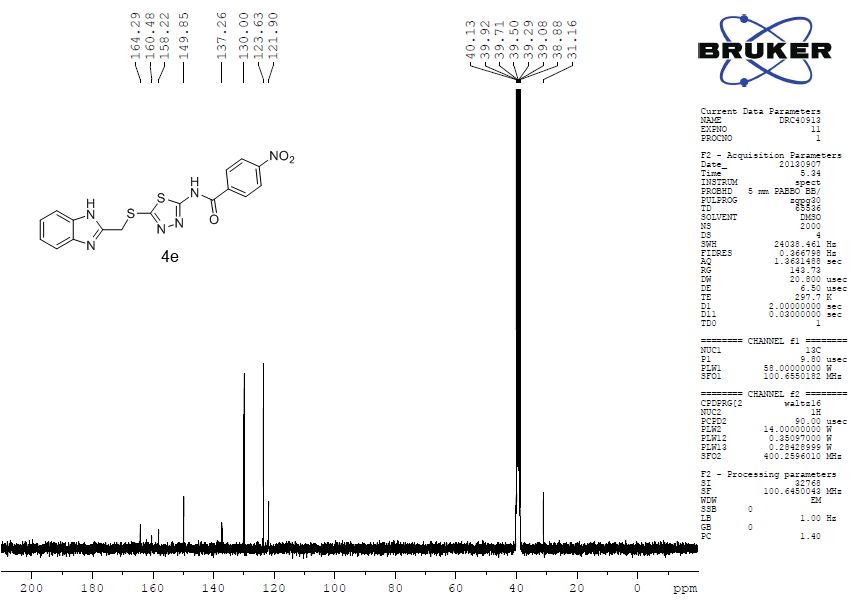

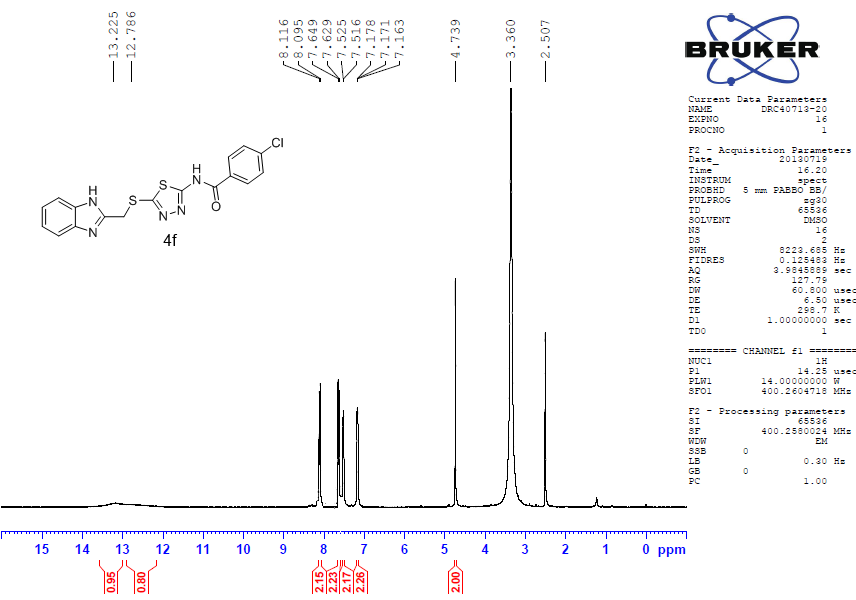

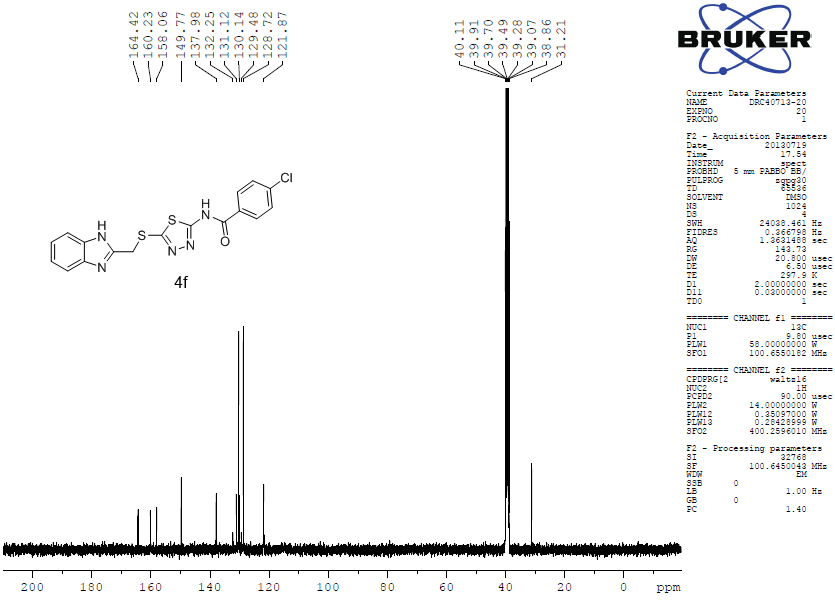

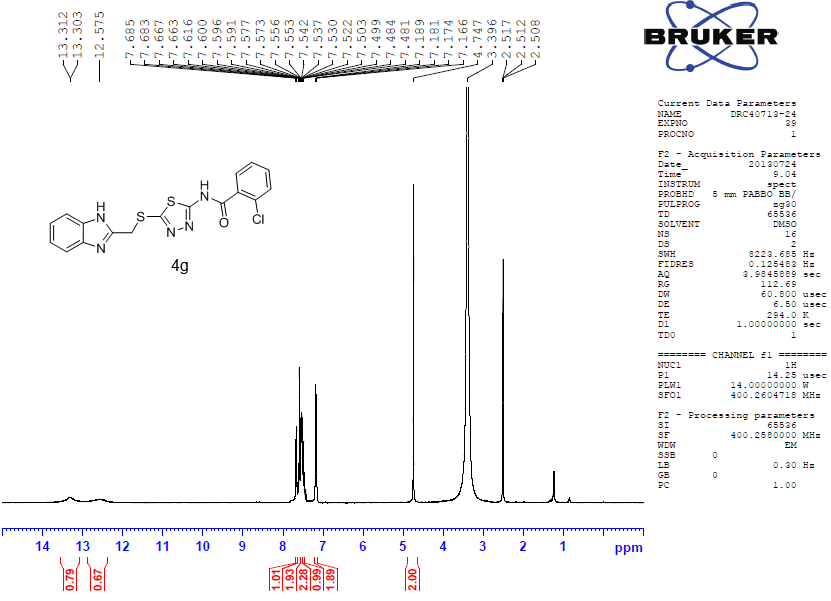

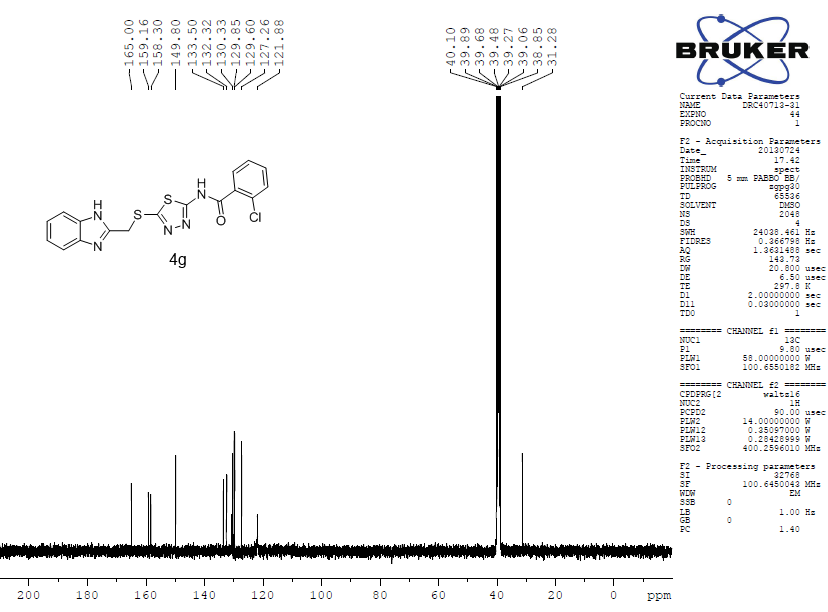

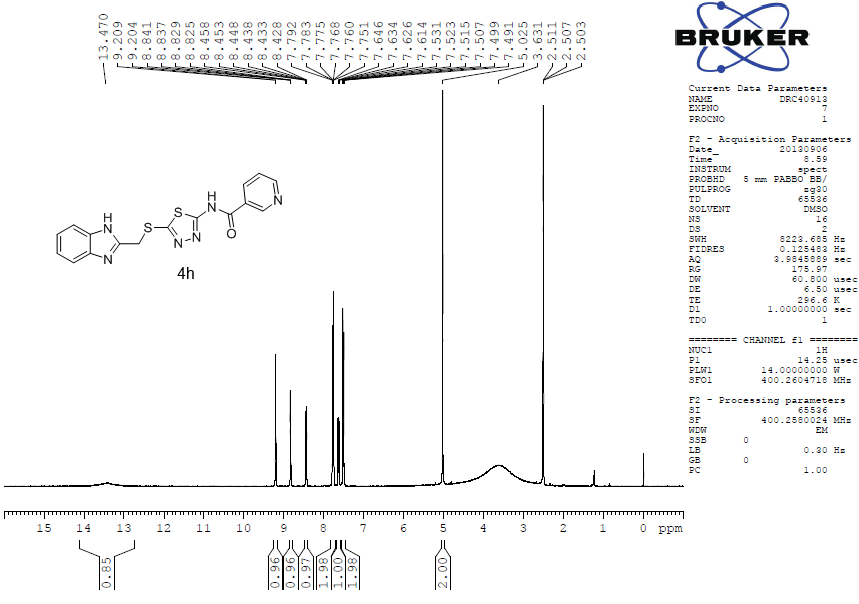

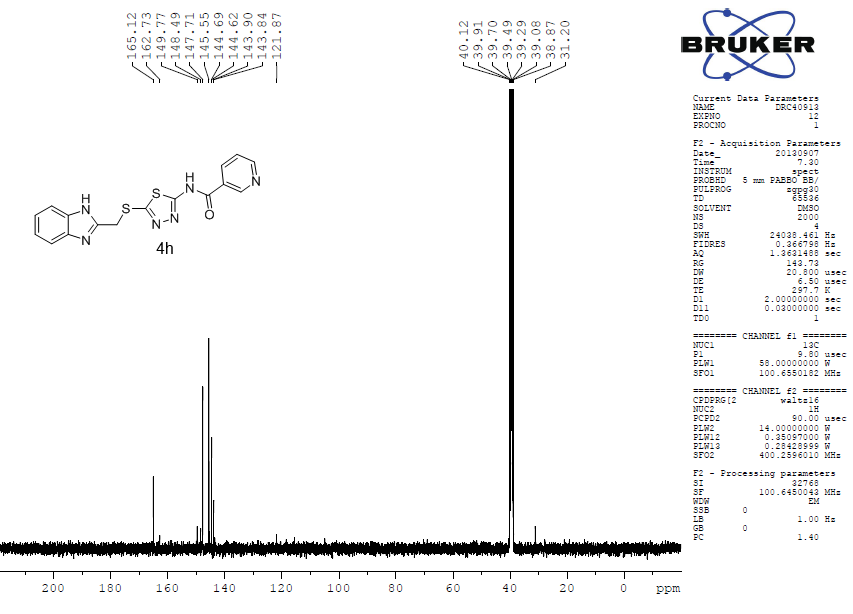

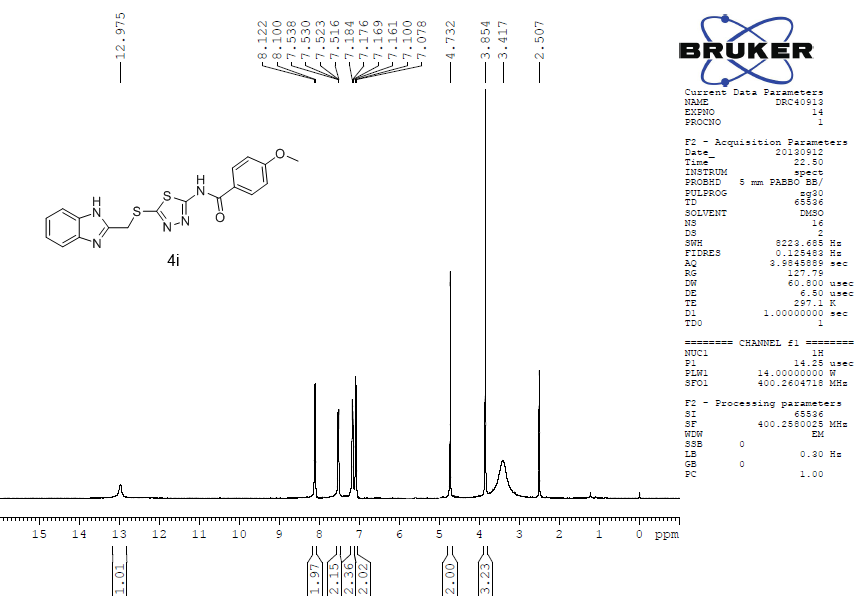

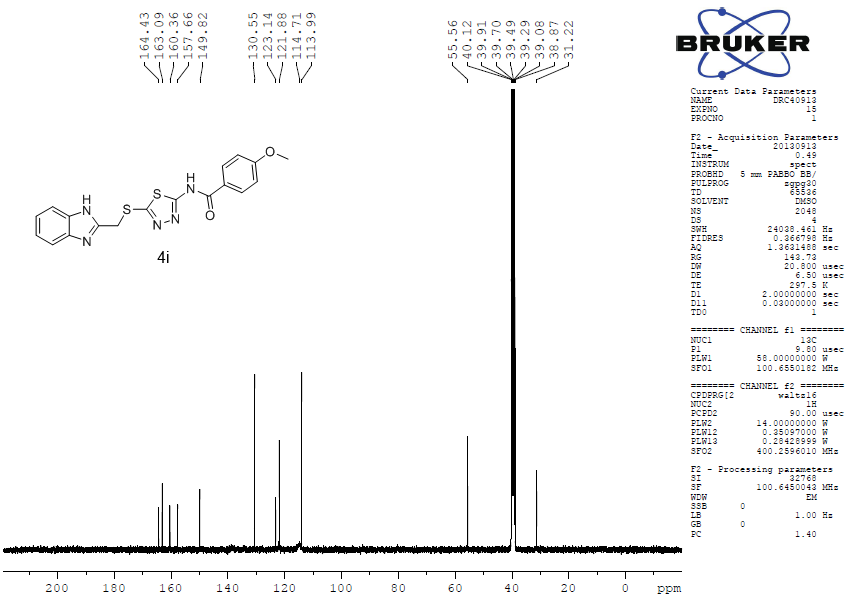

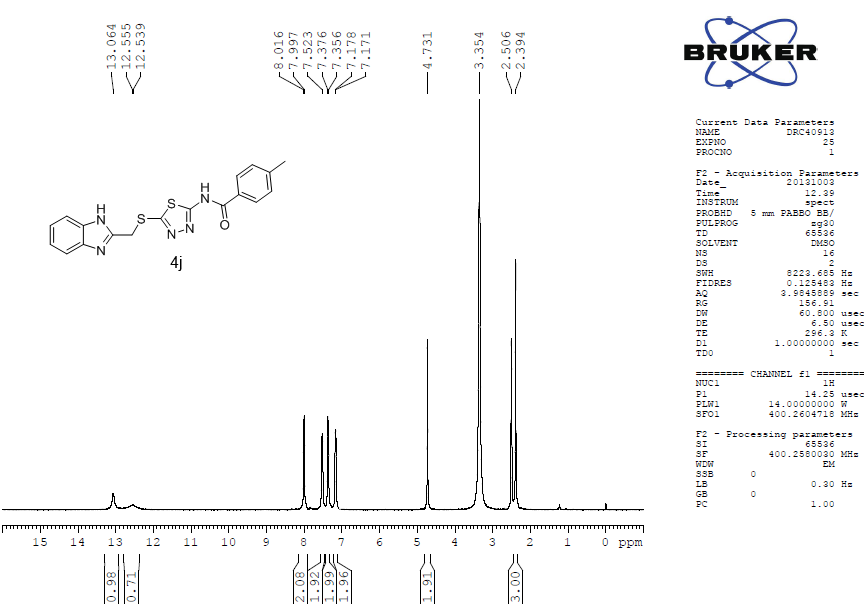

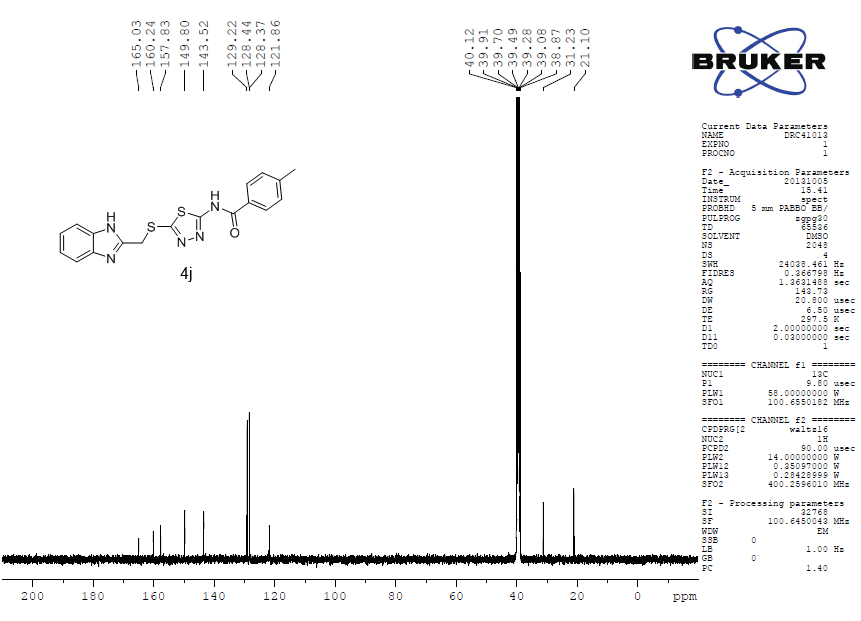

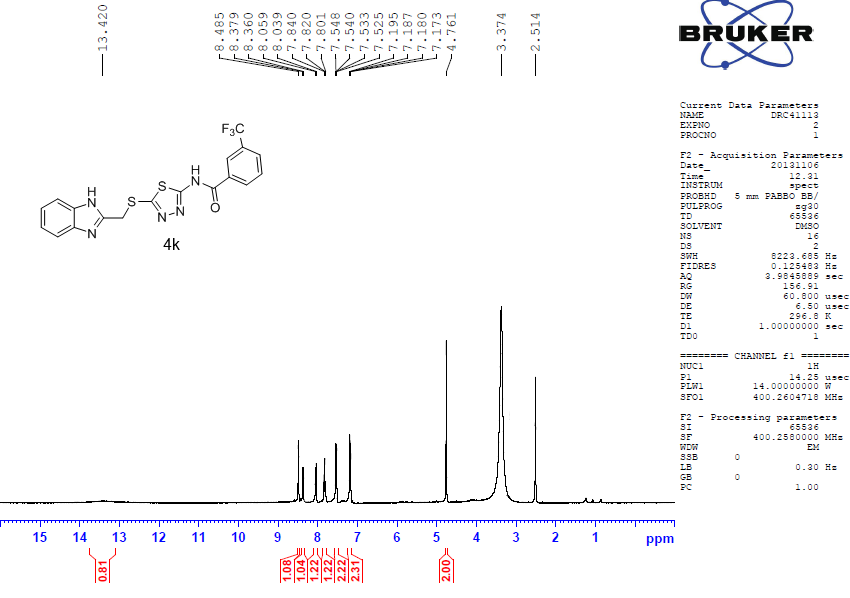

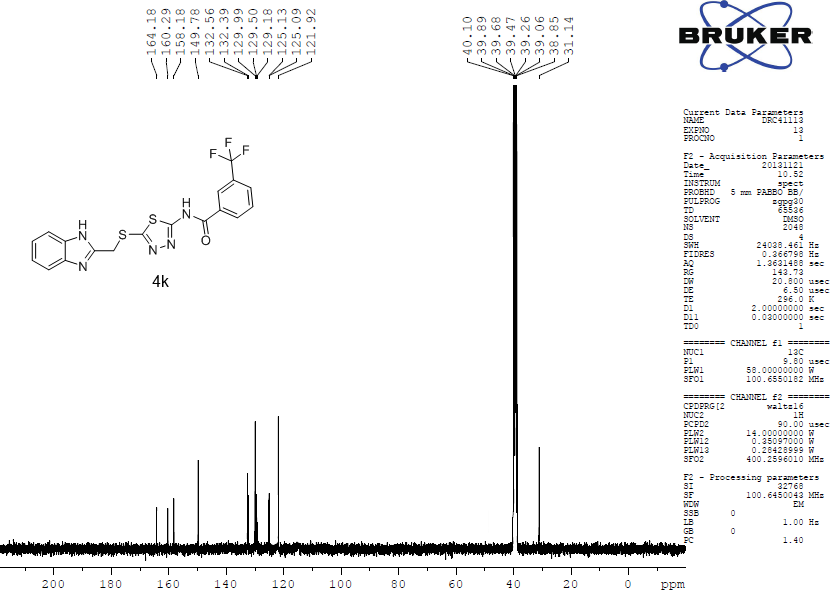

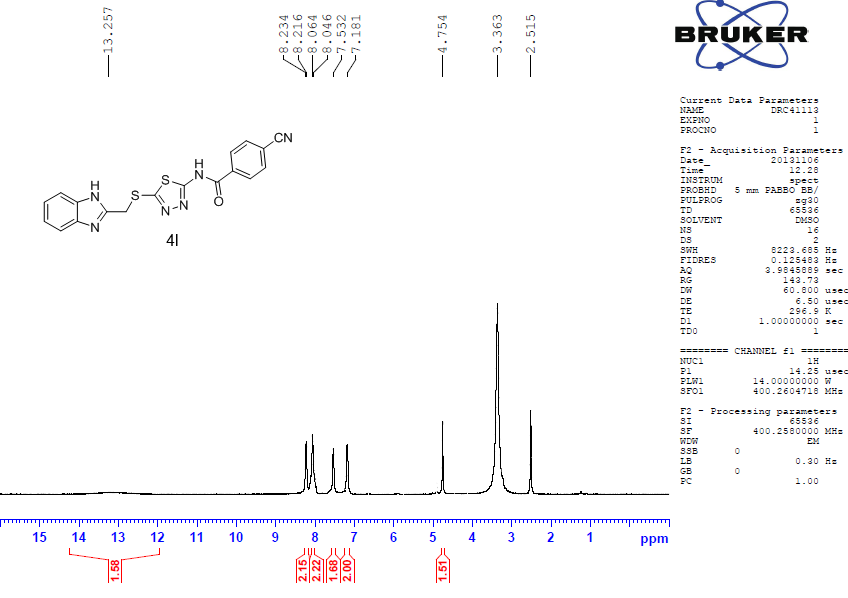

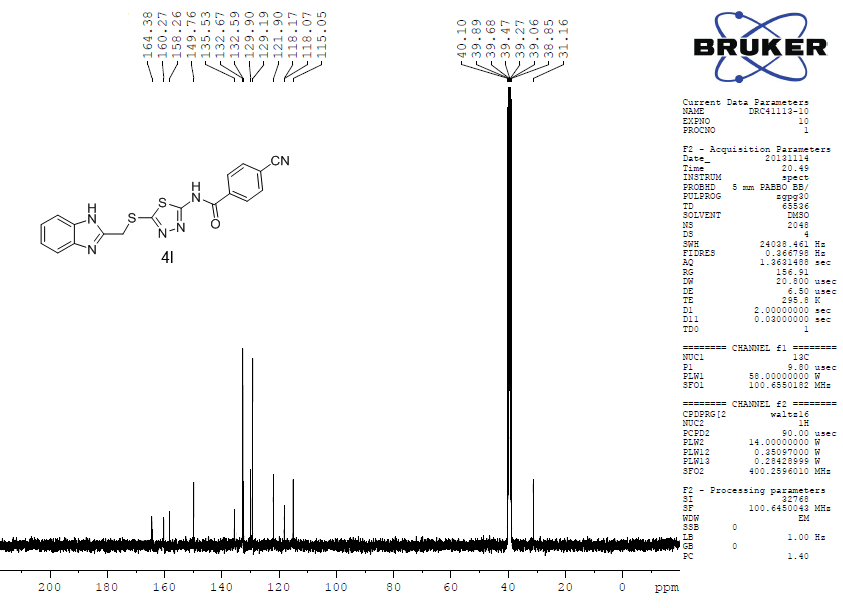

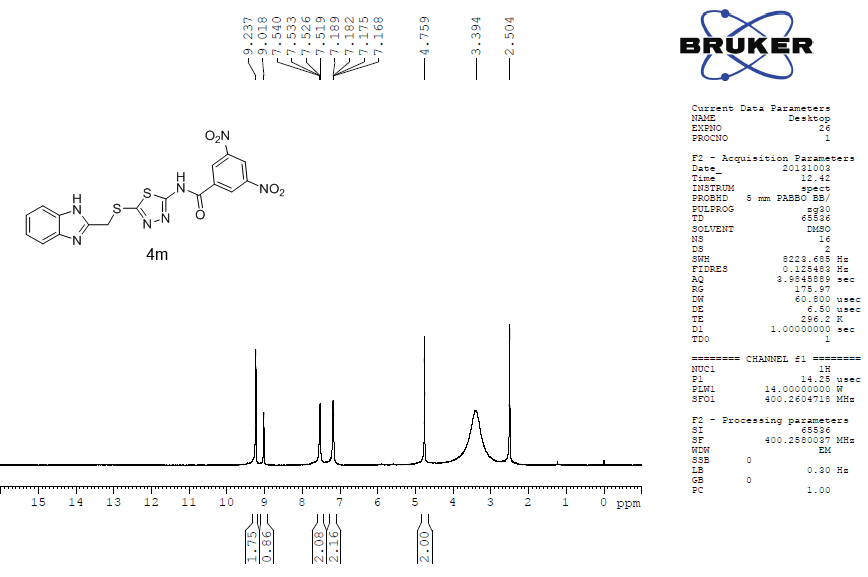

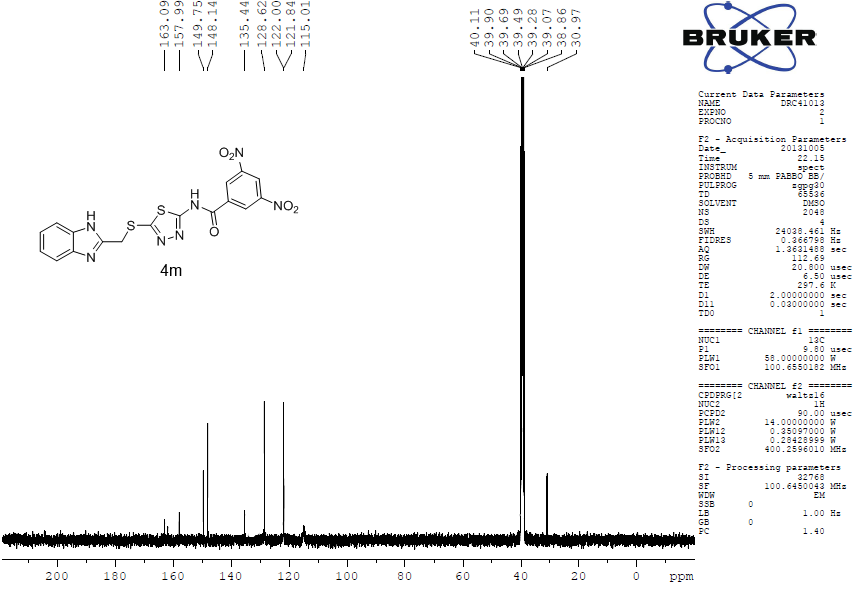

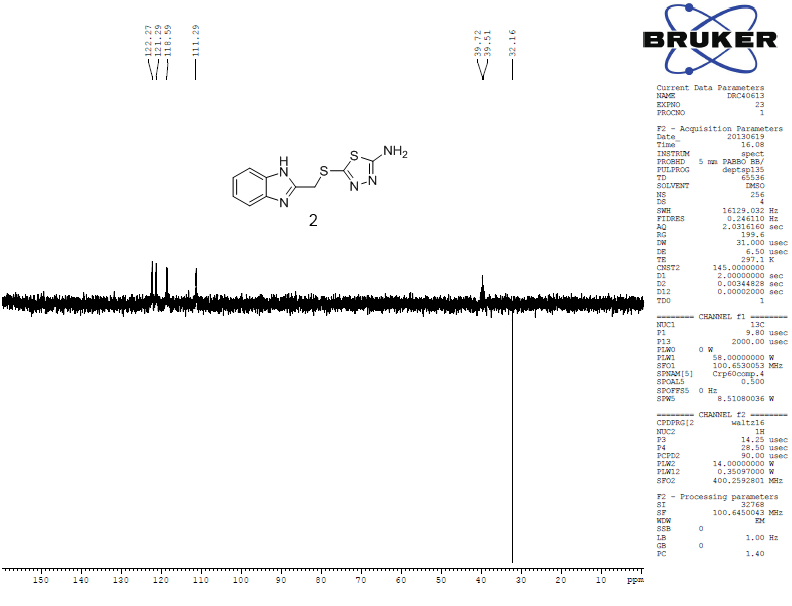

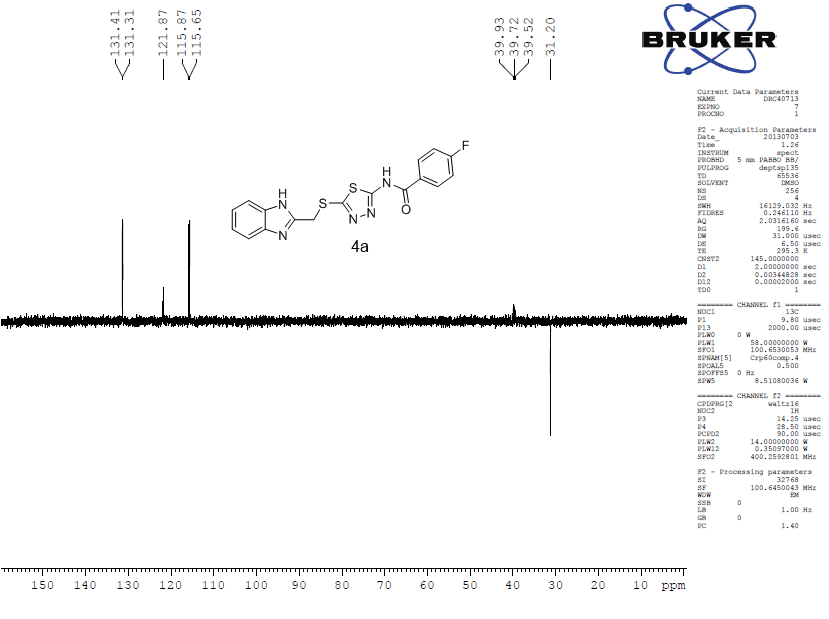

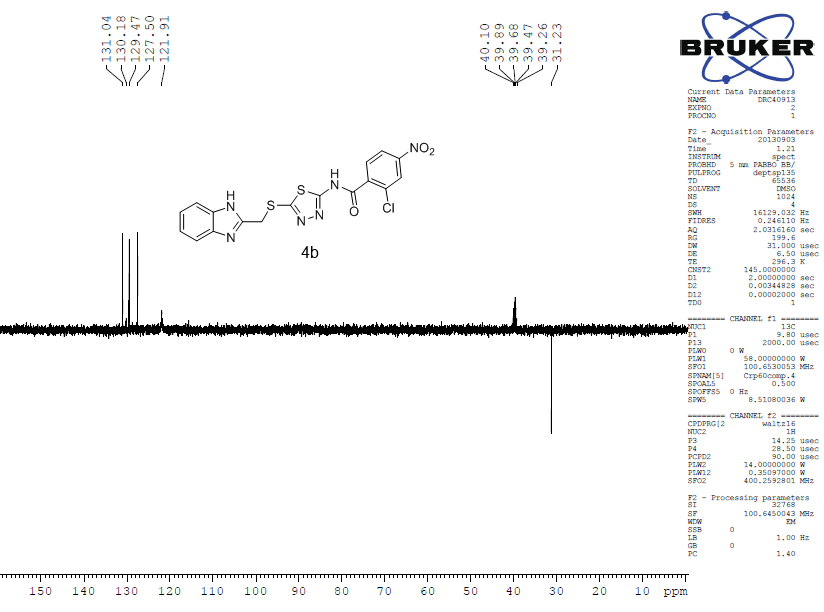

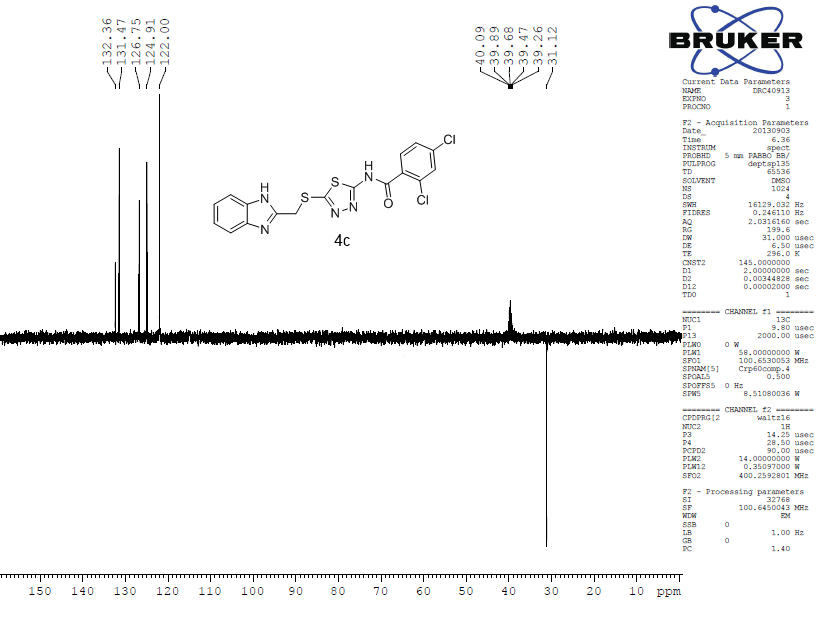

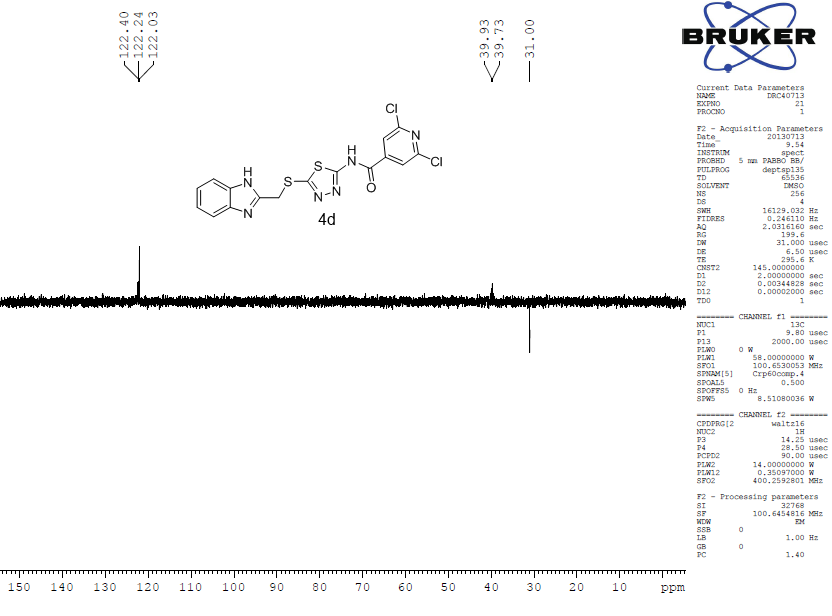

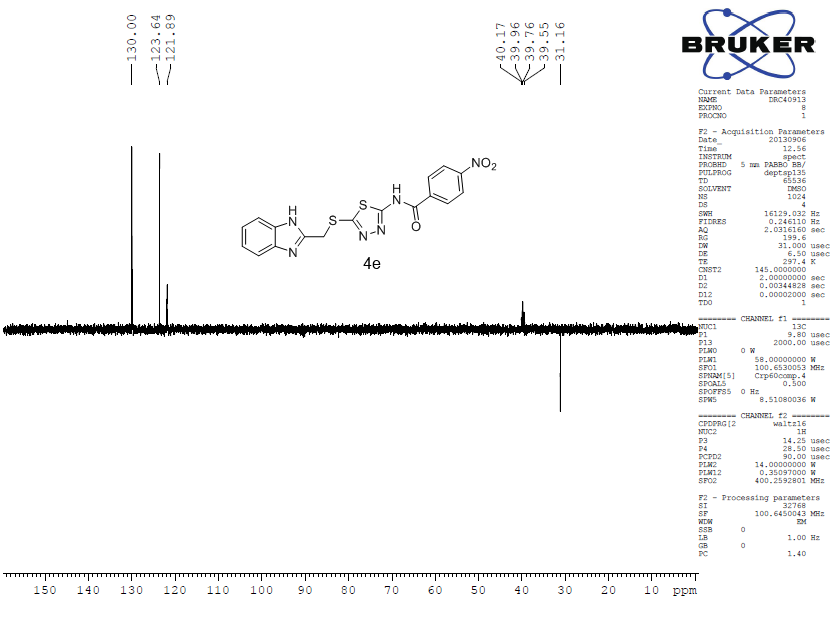

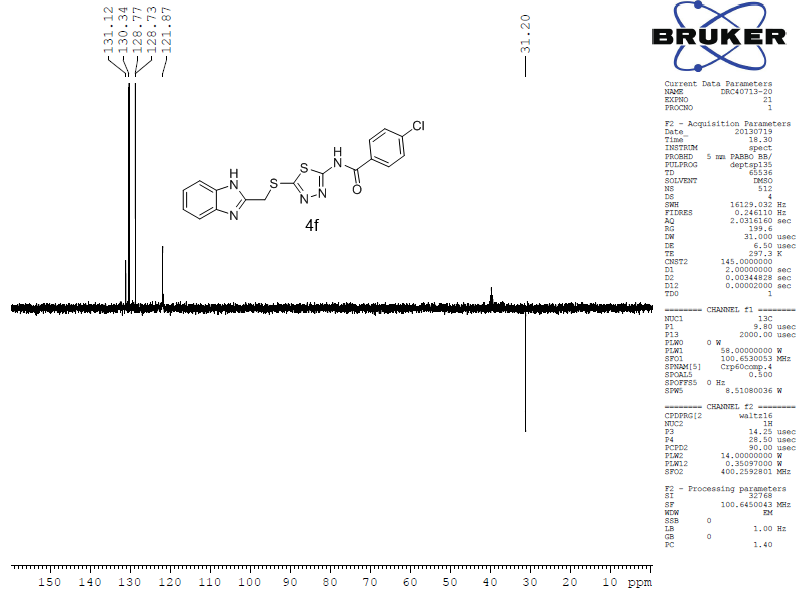

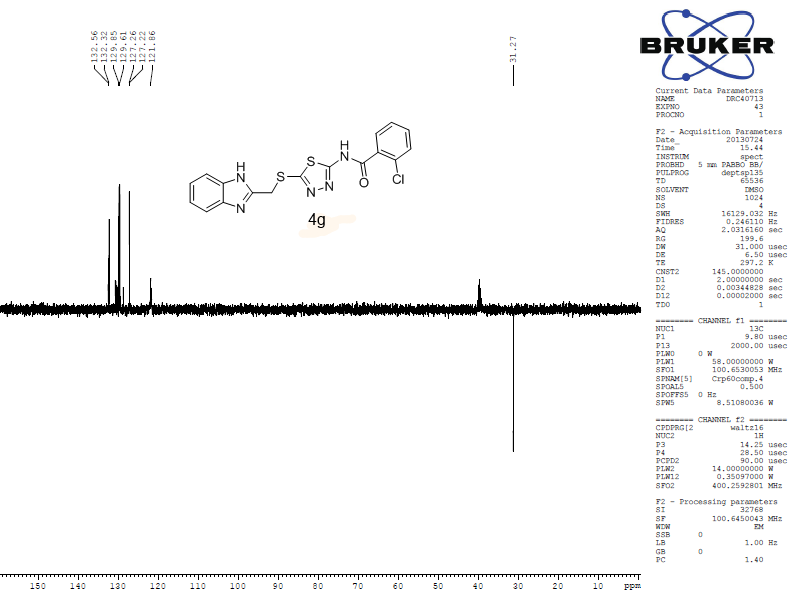

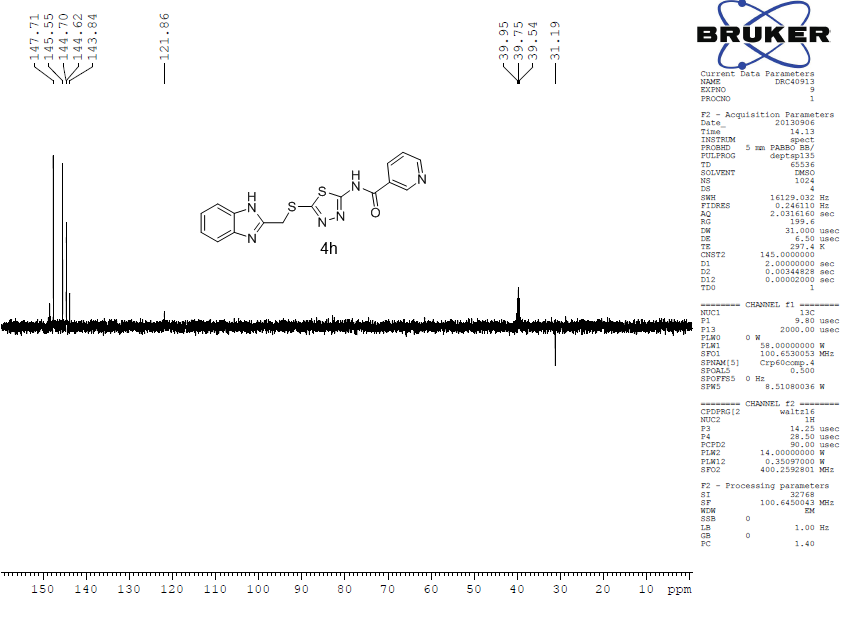

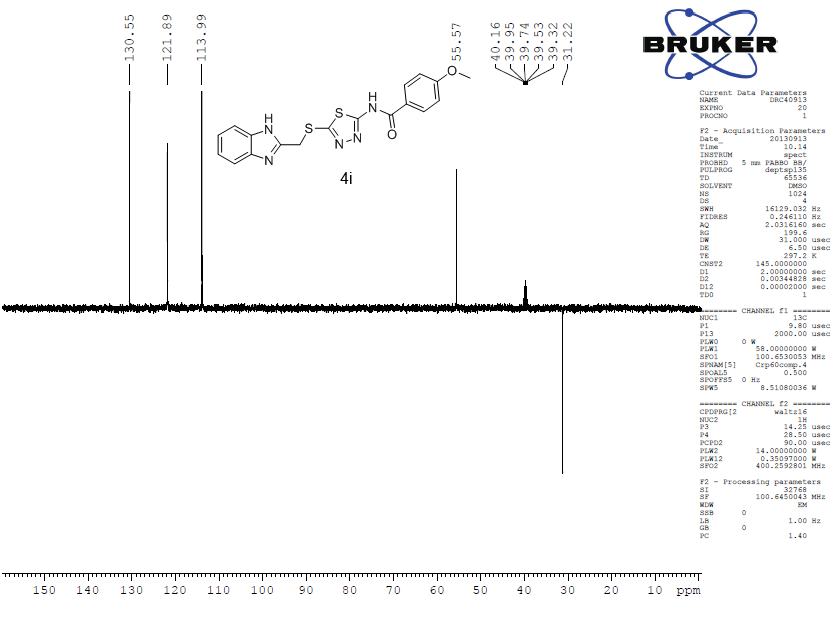

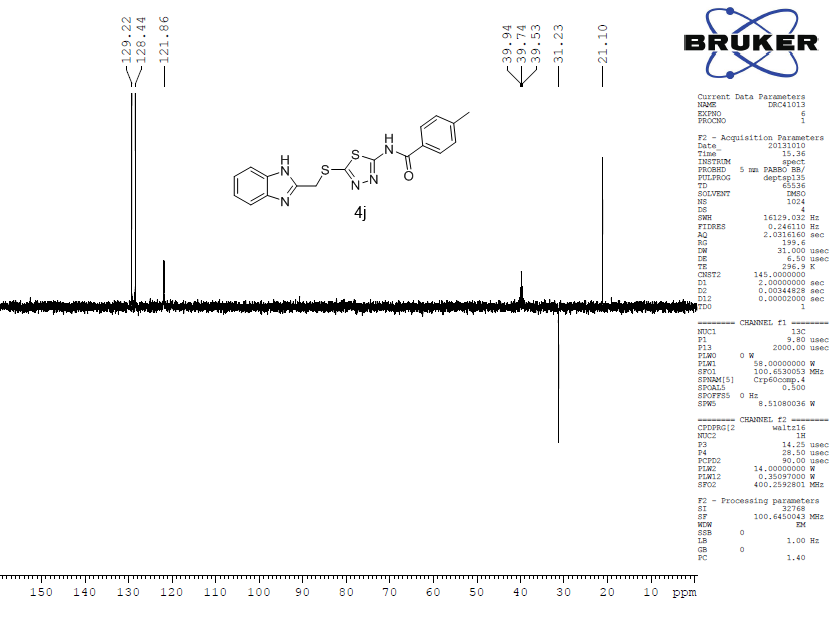

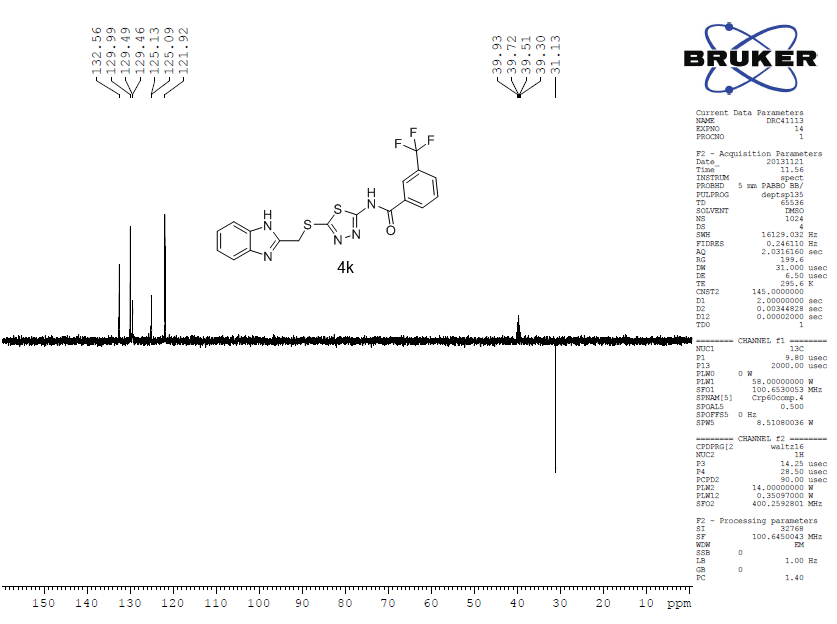

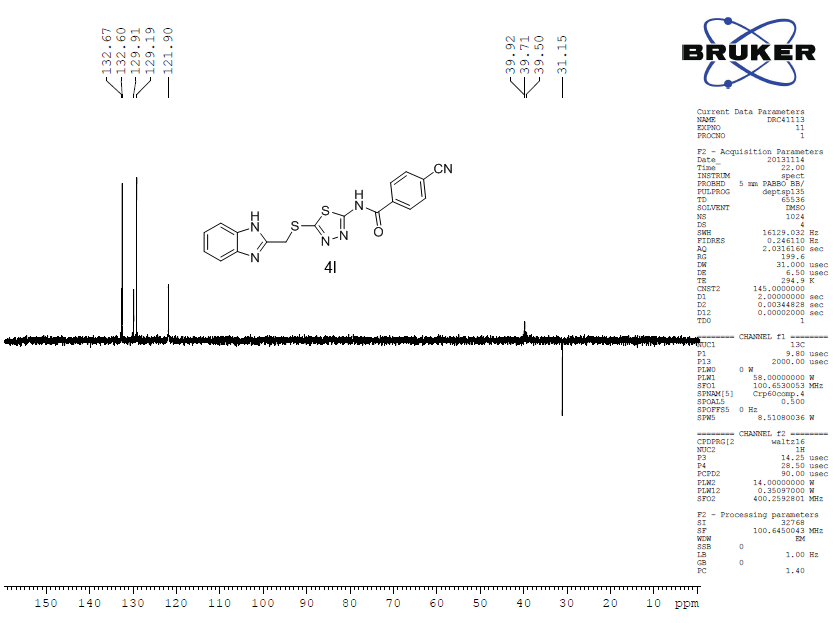

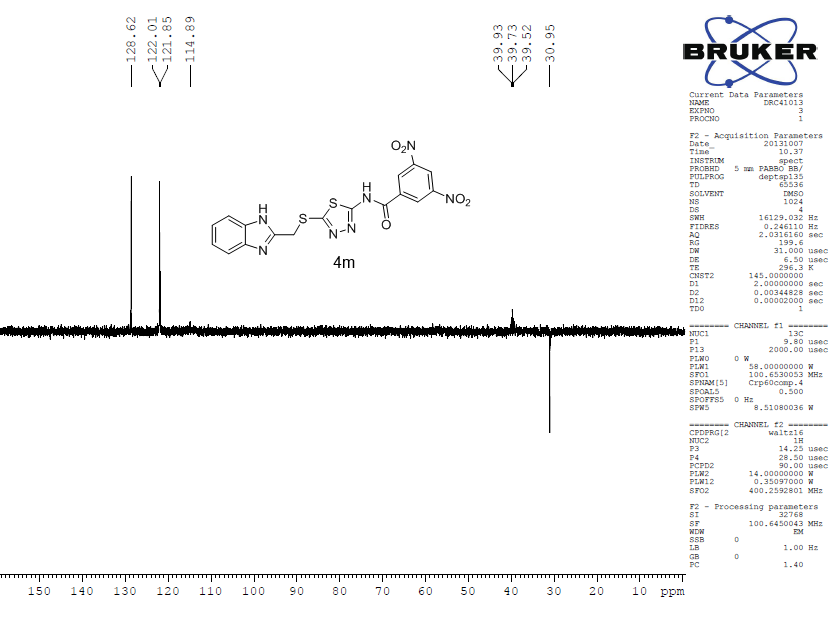


**References:**

1. Kharade P, Gaikwad D, Rathod S, Chougale U, Kadam S, Patil K, Choudhari P, Desai S. Assessing [DMAP-DABCO] AcO as an effective dual basic ionic liquid for the synthesis of chromeno [2, 3-d] pyrimidine derivatives and exploring their anti-cancer activity through computational analysis. J Mol Liq. 2024;416:126484. https://doi.org/10.1016/J.MOLLIQ.2024.126484.
2. Patil P, Nerlekar N, Rathod S, Mhaldar P, Najm T, Bansode P, Jadhav J, Dandge P, Choudhari P, Pore D, Rashinkar G. Novel sulphonamide-azaheterocycle conjugates and their anti-cancer, anti-inflammatory, anti-diabetic, anti-angiogenesis activity and molecular docking studies. Results Chem. 2024;7:101476. https://doi.org/10.1016/j.rechem.2024.101476.
3. Rathod S, Dey S, Pawar SP, Dhavale R, Choudhari E, Rajakumara E, Mahuli D, Bhagwat D, Tamboli Y, Sankpal P, Mali S. Identification of potential biogenic chalcones against antibiotic resistant efflux pump (AcrB) via computational study. J Biomol Struct Dyn. 2024;42:5178-5196. https://doi.org/10.1080/07391102.2023.2225099.
4. Prudent R, Moucadel V, Nguyen CH, Barette C, Schmidt F, Florent JC, Lafanechere L, Sautel CF, Duchemin-Pelletier E, Spreux E, Filhol O. Antitumor activity of pyridocarbazole and benzopyridoindole derivatives that inhibit protein kinase CK2. Cancer Res. 2010;70:9865-9874. https://doi.org/10.1158/0008-5472.can-10-0917.
5. Basha GM, Parulekar RS, Al-Sehemi AG, Pannipara M, Siddaiah V, Kumari S, Choudhari PB, Tamboli Y. Design and in Silico Investigation of Novel Maraviroc Analogues as Dual Inhibition of CCR-5/SARS-CoV-2 Mpro. J Biomol Struct Dyn. 2022;40:11095–11110. https://doi.org/10.1080/07391102.2021.1955742.
6. Shivakumar D, Williams J, Wu Y, Damm W, Shelley J, Sherman W. Prediction of Absolute Solvation Free Energies Using Molecular Dynamics Free Energy Perturbation and the Opls Force Field. J Chem Theory Comput. 2010;6:1509–1519. https://doi.org/10.1021/ct900587b.
7. Daina A, Michielin O, Zoete V. SwissADME: a free web tool to evaluate pharmacokinetics, drug-likeness and medicinal chemistry friendliness of small molecules. Sci Rep. 2017;7:42717. <https://doi.org/10.1038/srep42717>.
8. Dassault Systèmes, BIOVIA Discovery Studio Visualizer, (2020).
9. Dallakyan S, Olson AJ. Small-molecule library screening by docking with PyRx. Methods Mol Biol. 2015;1263:243–250. <https://doi.org/10.1007/978-1-4939-2269-7_19>.
10. Daina A, Michielin O, Zoete V. SwissADME: a free web tool to evaluate pharmacokinetics, drug-likeness and medicinal chemistry friendliness of small molecules. Sci Rep. 2017;7:42717. <https://doi.org/10.1038/srep42717>.
11. Pires DE, Blundell TL, Ascher DB. pkCSM: Predicting Small-Molecule Pharmacokinetic and Toxicity Properties Using Graph-Based Signatures. J Med Chem. 2015;58:4066–4072. <https://doi.org/10.1021/acs.jmedchem.5b00104>.
12. Neese F. The ORCA program system. Wiley Interdiscip Rev Comput Mol Sci. 2012;2:73–78. <https://doi.org/10.1002/wcms.81>.
13. Snyder HD, Kucukkal TG. Computational Chemistry Activities with Avogadro and ORCA. J Chem Educ. 2021;98:1335–1341. <https://doi.org/10.1021/acs.jchemed.0c00959>.
14. Salunkhe SY, Gurav RP, Rathod SS, Choudhari PB, Yadav TP, Wakshe SB, Anbhule PV, Kolekar GB. Biological evaluation, molecular modeling and dynamic simulation of IDQ bulk and IDQNPs: Organo nano-bio interface in the medical field. J Mol Struct. 2024;1301:137288. <https://doi.org/10.1016/j.molstruc.2023.137288>.
15. Kharade PR, Chougale UB, Gaikwad DS, Kadam SS, Patil KN, Rathod SS, Choudhari PB, Desai SS. Synthesis and in vitro evaluation of tetrahydropyridines as potential CDK2 and DprE1 inhibitors. Res Chem Intermed. 2024;50:1777–1808. <https://doi.org/10.1007/s11164-024-05228-2>.
16. Berman HM, Westbrook J, Feng Z, Gilliland G, Bhat TN, Weissig H, Shindyalov IN, Bourne PE. The Protein Data Bank. Nucleic Acids Res. 2000;28:235. <https://doi.org/10.1093/nar/28.1.235>.
17. Eberhardt J, Santos-Martins D, Tillack AF, Forli S. AutoDock Vina 1.2.0: New Docking Methods, Expanded Force Field, and Python Bindings. J Chem Inf Model. 2021;61:3891–3898. <https://doi.org/10.1021/acs.jcim.1c00203>.
18. Nguyen NT, Nguyen TH, Pham TNH, Huy NT, Van Bay M, Pham MQ, Nam PC, Vu VV, Ngo ST. Autodock Vina Adopts More Accurate Binding Poses but Autodock4 Forms Better Binding Affinity. J Chem Inf Model. 2020;60:204–211. https://doi.org/10.1021/acs.jcim.9b00778
19. Schrödinger, Desmond molecular dynamics system. Maestro-Desmond interoperability tools, D. E. Shaw Research (2019).
20. E. Chow, C.A. Rendleman, K.J. Bowers, R.O. Dror, D.H. Hughes, J. Gullingsrud, F.D. Sacerdoti, D.E. Shaw, Desmond Performance on a Cluster of Multicore Processors Hardware and Operating Environment Benchmark Systems and Simulation Parameters, DE Shaw Research Technical Report DESRES/TR--2008-01 (2008).
21. Choudhari S, Patil SK, Rathod S. Identification of hits as anti-obesity agents against human pancreatic lipase via docking, drug-likeness, in-silico ADME(T), pharmacophore, DFT, molecular dynamics, and MM/PB(GB)SA analysis. J Biomol Struct Dyn. 2023;1–23. <https://doi.org/10.1080/07391102.2023.2258407>.
22. Rathod S, Shinde S, Choudhari P, Sarkate A, Chaudhari S, Shingan A. Exploring binding potential of two new indole alkaloids from Nauclea officinalis against third and fourth generation EGFR: drug-likeness, in silico ADMET, docking, DFT, molecular dynamics simulation, and MMGBSA study. Nat Prod Res. 2024. <https://doi.org/10.1080/14786419.2023.2301678>.
23. Bowers KJ, Chow E, Xu H, Dror RO, Eastwood MP, Gregersen BA, Klepeis JL, Kolossvary I, Moraes MA, Sacerdoti FD, Salmon JK, Shan Y, Shaw DE. Scalable algorithms for molecular dynamics simulations on commodity clusters. Proceedings of the 2006 ACM/IEEE Conference on Supercomputing, SC’06. 2006. <https://doi.org/10.1145/1188455.1188544>.
24. W.L. Jorgensen, J. Chandrasekhar, J.D. Madura, R.W. Impey, M.L. Klein, Comparison of simple potential functions for simulating liquid water, J Chem Phys 79 (1983) 926–935. <https://doi.org/10.1063/1.445869>.
25. Shivakumar D, Williams J, Wu Y, Damm W, Shelley J, Sherman W. Prediction of absolute solvation free energies using molecular dynamics free energy perturbation and the OPLS force field. J Chem Theory Comput. 2010;6:1509–1519. <https://doi.org/10.1021/ct900587b>.
26. Shinde SS, Sarkate AP, Rathod SS, Kilbile JT, Chaudhari SY, Yadala R, Pawar SC, Wakte PS. Synthesis, biological evaluation, and computational studies of thiazolyl hydrazone derivatives as triple mutant allosteric EGFR inhibitors. J Chin Chem Soc. 2024;71:706–720. <https://doi.org/10.1002/JCCS.202400084>.
27. Martyna GJ, Klein ML, Tuckerman M. Nosé–Hoover chains: The canonical ensemble via continuous dynamics. J Chem Phys. 1992;97:2635–2643. <https://doi.org/10.1063/1.463940>.
28. Indumathi T, Muthusankar A, Shanmughavel P, Rajendra Prasad KJ. Synthesis of hetero annulated carbazoles: exploration of in vitro cytotoxicity and molecular docking studies. Med Chem Commun. 2013;4:450–455. <https://doi.org/10.1039/C2MD20335A>
